# Efficacy of ultrasound-guided external oblique intercostal plane block in laparoscopic common bile duct exploration: A double-blinded randomized controlled study

**DOI:** 10.1371/journal.pone.0355377

**Published:** 2026-08-06

**Authors:** Qianqian Jia, Lili Zhang, Yang Han, Yating Yang, Qian Sun, Zhuo Liu, Chenxi Wu, Qiujun Wang, Fei Liu, Shujuan Liang

**Affiliations:** 1 Department of Anesthesiology, The First Hospital of Qinhuangdao, Qinhuangdao, Hebei, China; 2 General Practice Department, The First Hospital of Qinhuangdao, Qinhuangdao, Hebei, China; 3 Department of Anesthesiology, Hebei Medical University Third Hospital, Shijiazhuang, Hebei, China; 4 Arthroscopy Department, The First Hospital of Qinhuangdao, Qinhuangdao, Hebei, China; E-Da Cancer Hospital, TAIWAN

## Abstract

**Background:**

Ultrasound-guided external oblique intercostal plane block (EOIPB) is a modified block technique used in the past few years for anterolateral upper abdominal wall analgesia. This study observed the efficacy of EOIPB in laparoscopic common bile duct exploration (LCBDE).

**Objectives:**

To evaluate the efficacy of EOIPB in patients undergoing LCBDE.

**Methods:**

Sixty patients undergoing elective LCBDE were randomly assigned to two groups, an EOIPB group and a control group. In the EOIPB group, the patients received ultrasound-guided right EOIPB with a total of 20 mL of 0.5% ropivacaine before anesthesia induction. In the control group, the patients received no intervention before anesthesia induction. The primary outcomes were visual analog scale (VAS) scores within 24 h postoperatively and the number of patient-controlled analgesia (PCA) uses 24 h postoperatively. The secondary outcomes were dermatomal coverage of EOIPB, intraoperative vital signs, the intraoperative dose of remifentanil, and the 24-h postoperative quality of recovery-40 (QoR-40) score of the two groups.

**Results:**

In the EOI group, VAS scores at rest and movement were significantly lower than in the control group at 30 min, 6 h, and 12 h postoperation (*p* < 0.05), with no significant difference at 24 h (*p* > 0.05). The number of PCA uses within 24 h was significantly lower in the EOIPB group (1.60 ± 0.89) compared to the control group (2.50 ± 1.11, *p* < 0.05). Dermatomal coverage in the EOIPB group achieved 100% T6–T10 at the anterior median and midclavicular lines, 100% T6–T9 at the anterior axillary line, and 93.3% T7–T8 at the posterior axillary line. Mean arterial pressure (MAP) was significantly lower in the EOIPB group at skin incision (T2), 10 min postoperative (T3), and 30 min after extubation (T5) (*p* < 0.05), and heart rate (HR) was significantly lower at T2 (*p* < 0.05). The intraoperative remifentanil dose was significantly reduced in the EOIPB group (0.84 ± 0.13 mg) compared to the control group (0.94 ± 0.14 mg, *p* < 0.05). The EOIPB group had a shorter bedridden time (23.33 ± 2.81 h vs. 25.23 ± 3.64 h, *p* < 0.05) and a higher QoR-40 score (170.6 ± 5.2 vs. 165.5 ± 6.3, *p* < 0.05), indicating improved recovery.

**Conclusion:**

The use of EOIPB in LCBDE can reduce VAS scores and the number of PCA uses 24 h postoperatively. EOIPB also steadied the intraoperative vital signs, reduced the dose of remifentanil, and improved the quality of recovery. EOIPB can be safely and effectively used in LCBDE.

## 1. Background

Laparoscopic common bile duct exploration (LCBDE) is a safe, reliable, cost-effective, and common treatment for common bile duct stones [1,2]. However, postoperative pain stimulation can easily lead to pain, tension, anxiety and other adverse emotions in patients, which affects the surgical outcome and prognosis and prolongs the hospital stay; therefore, effective analgesia is essential for patients undergoing LCBDE [3].

Opioids or epidural analgesia are the first choice for postoperative abdominal wall analgesia. However, they are associated with risks of hypotension, urinary retention, nausea, vomiting, and respiratory depression [4–5]. External oblique intercostal plane block (EOIPB) is a recent modified block technique that blocks the lateral and anterior cutaneous branches of the intercostal nerves from T6/7 to T10/11 for analgesia of the anterolateral upper abdominal wall [6]. Compared to other analgesia options, EOIPB is safer, simpler, less time-consuming, and has fewer complications [7]. Although some case series have demonstrated that EOIPB provides effective analgesia in upper abdominal surgeries [6,7], there is still a lack of randomized studies examining LCBDE. The study aimed to observe the efficacy of EOIPB in LCBDE.

## 2. Methods

### 2.1. Study design

This study was carried out at the First Hospital of Qinhuangdao from March 2023 to February 2024 and was approved by the Ethics Committee of the First Hospital of Qinhuangdao (ID: 2023KZ077). The study was registered on ClinicalTrials.gov (NCT06684210, registration date: 17/10/2024). It adheres to the principles outlined in the Declaration of Helsinki and the CONSORT 2010 guidelines. Informed consent was obtained from all enrolled patients concerning study enrollment, anonymized data collection, and publication.

Sixty patients who underwent LCBDE were enrolled in the study. Inclusion criteria were as follows: aged 18–65 years; American Society of Anesthesiologists (ASA) physical status I–II; diagnosis of choledocholithiasis and scheduled for elective LCBDE; clear consciousness; ability to cooperate with postoperative pain scoring and follow-up; and provision of written informed consent. Exclusion criteria were as follows: history of liver disease, alcoholism, or drug abuse; mental illness; prior abdominal surgery or trauma; long-term opioid use; benign biliary stricture, biliary tract malignancy, ampullary lesions, or other malignant obstructive diseases; coagulation disorders; systemic or local infection at the injection site; conversion to open surgery during the procedure; or allergy to local anesthetics. This trial used assessor–statistician blinding. All patients were evaluated preoperatively by an anesthesiologist. A research assistant (not involved in the study intervention, anesthesia management, or data collection) generated random numbers using a random number table. Patients were randomized to either the EOIPB group or the control group, using a closed, sequentially numbered opaque envelope method. The research assistant opened the envelopes and assigned patients to groups, and the allocation of the grouping result was disclosed only to the physician responsible for performing EOIPB. The anesthesiologists, statistician, and observers remained blinded to group allocation until the study was completed. During the preoperative visit, the observer instructed all patients on visual analog scale (VAS) use and trained them in the use of a patient-controlled analgesia (PCA) device for postoperative pain management. The observer remained outside the operating room during EOIPB and induction and was recalled to the operating room before the procedure.

**Fig 1:**
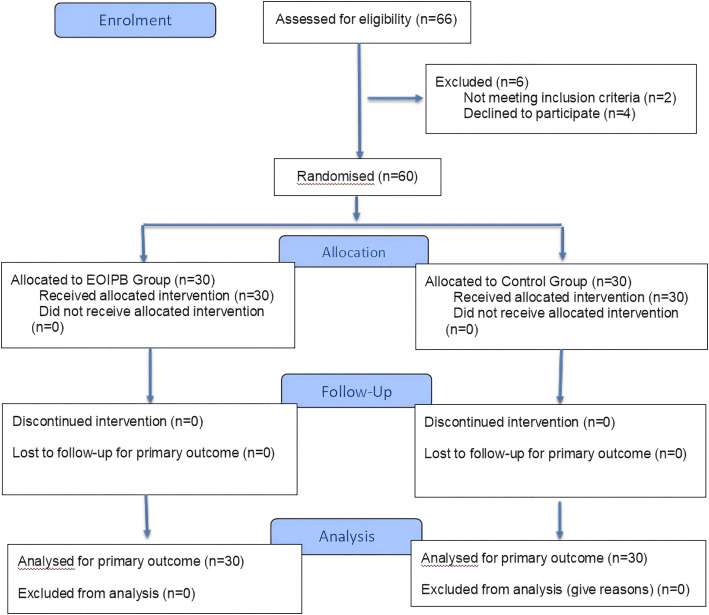
CONSORT flow diagram.

Of the 66 patients assessed for study eligibility, two did not meet the inclusion criteria and four were unwilling to participate. Finally, 60 patients were enrolled in the study. The study was completed with the 60 patients (Fig 1).

**Fig 2:**
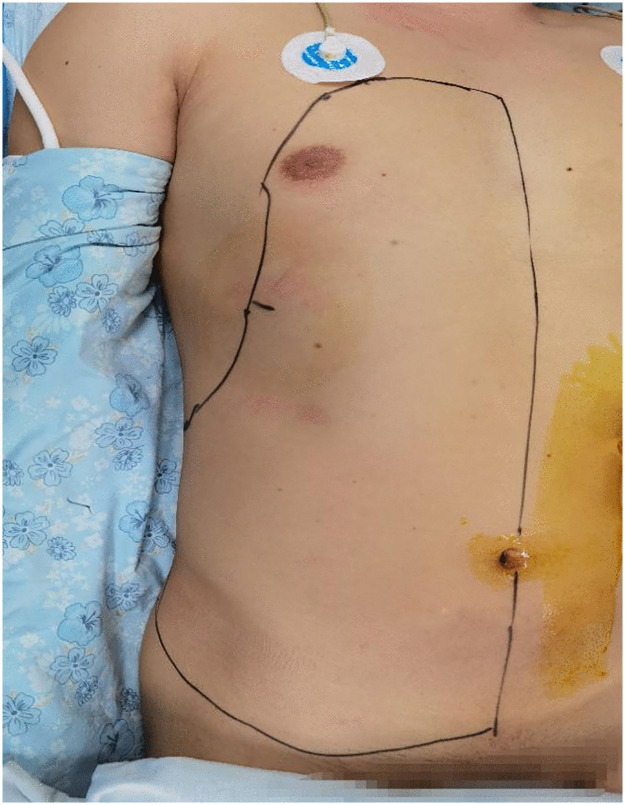
The dermatomal coverage of EOIPB.

The EOIPB was performed in the pre-anesthesia room. In the EOIPB group, a right-sided EOIPB was administered. The patient was observed for complications, such as hemorrhage, infection, nerve injury, allergic reactions, and pneumothorax. The block plane was tested 15 min after the procedure (Fig 2). The control group did not receive any intervention before induction. All patients were then transferred from the pre-anesthesia room to the operating room.

### 2.2. EOIPB administration

**Fig 3:**
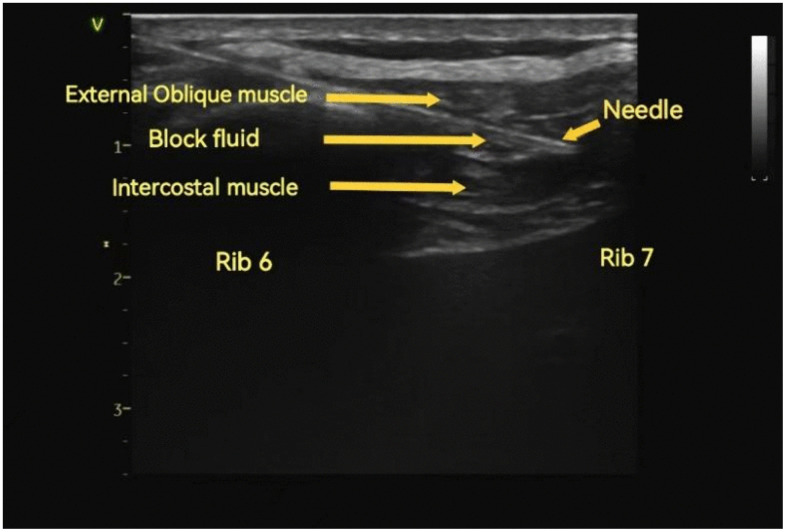
An ultrasonographic image illustrating the intervention. Blocking fluid was injected between the external oblique and intercostal muscles between the sixth and seventh ribs.

The patients were placed in the supine position with the ipsilateral arm abducted. A linear array probe (4–12 MHz, Vivid iq, GE, USA) was placed in a cephalad-to-caudad sagittal plane along the anterior axillary line at the level of the right sixth and seventh ribs, aligned with the xiphoid process, with the probe marker oriented cephalad. The probe was oriented inwards, perpendicular to the ribs. We then identified the structures from superficial to deep: subcutaneous tissue, external oblique muscle, intercostal muscles, pleura, and lung. A 20-gauge 80-mm needle (Dean, Shandong, China) was advanced from the cephalad to caudad direction until the tip was positioned between the external oblique and intercostal muscles between the sixth and seventh ribs. Then, 20 mL of 0.5% ropivacaine was injected using an in-plane technique (Fig 3). EOIPB was performed by a senior anesthesiologist with many years of plane block experience.

### 2.3. Anesthesia management

Upon arrival in the operating room, standard monitoring was initiated, including electrocardiography, noninvasive arterial blood pressure, and pulse oximetry. Anesthesia was induced with 0.05 mg/kg IV midazolam, 2 mg/kg IV propofol, 0.15 mg/kg IV *cis*-atracurium and 0.3 μg/kg sufentanil. Anesthesia was maintained using remifentanil at 0.1–0.2 μg/kg/min, propofol at 4–6 mg/kg/h, and 5 mg of IV *cis*-atracurium intermittently. Bispectral index (BIS) was maintained at 40–60, and HR and mean arterial pressure (MAP) were within the baseline ± 20%. Remifentanil was administered at a basal infusion rate of 0.1 μg/kg/min. The intraoperative infusion rate was adjusted based on heart rate (HR), MAP, sweating, lacrimation, coughing, and body movement. If pain-related responses occurred, the infusion rate was increased by 0.05 μg/kg/min. If bradycardia or hypotension occurred, the infusion rate was reduced by 0.05 μg/kg/min. After residual neuromuscular blockade was reversed, tracheal extubation was performed in patients with regular spontaneous breathing.

### 2.4. Postoperative analgesia

Postoperatively, 2 μg/kg sufentanil, 5 mg of tropisetron, and normal saline mixed to 100 mL (2 mL/h basal infusion; 1 mL IV bolus; 15 min locking) as PCIA was administered. Patients with VAS scores ≥4 or those requesting additional analgesia received PCA as rescue analgesia. If analgesic efficacy was suboptimal, PCA administration could be repeated after 15 min following physician evaluation to ensure adequate pain control. No additional analgesics were administered 24 h postoperatively. PCA use was documented, including dosage, timing, and frequency.Nurses in the post-anesthesia care unit and ward assessed patients’ VAS scores; all nurses were blinded to group allocation.

### 2.5. Surgical procedure

All procedures were performed by surgeons from the same surgical team using choledochoscopy-assisted LCBDE. The specific stone removal strategy was standardized as follows: after longitudinal choledochotomy of the common bile duct, a flexible choledochoscope was inserted for full exploration of the biliary tree. For most calculi, choledochoscope-guided basket retrieval was the preferred method of stone extraction. For small, sandy or residual calculi, balloon catheter extraction was used as an adjunctive technique to facilitate biliary tract clearance. No endoscopic sphincterotomy was performed in any of the enrolled patients to avoid postoperative biliary stricture, reflux cholangitis, and other complications caused by sphincter injury. Direct manual stone removal without choledochoscopy assistance was not applied in any case to ensure the thoroughness of stone clearance. For bile duct closure, a unified standardized suture method was adopted for all patients after confirming complete stone clearance and unobstructed biliary drainage. This method comprised continuous interrupted suturing of the common bile duct incision with 4−0 absorbable surgical sutures alongside the routine placement of abdominal drainage tube near the biliary tract incision. No T-tube drainage was performed in any of the cases. Carbon dioxide (CO_2_) pneumoperitoneum was established at an insufflation rate of 2–4 L/min. Intra-abdominal pressure was maintained at 12–14 mmHg and dynamically adjusted by the anesthesiologist based on the patient’s vital signs (blood pressure, heart rate, airway pressure) and surgical requirements.

The surgical incisions for LCBDE were located at the umbilicus (observation port, 1.0 cm), the right subcostal region at the midclavicular line (primary operating port, 1.5–2.0 cm), the subxiphoid (auxiliary port, 0.5 cm), and the right inferolateral costal region (second auxiliary port, 0.5 cm). All incisions underwent a standardized fascial closure protocol at the completion of surgery: the fascial layer was closed with interrupted sutures using 4−0 absorbable sutures, and the skin layer was closed with intradermal sutures. Standardized fascial closure protocol included: interrupted suturing with uniform needle spacing and margin distances; avoidance of excessive tightness or looseness; suturing performed by the same surgical team with consistent suture tension and tissue layers; avoidance of repeated puncture, and excessive traction on the fascia and subcutaneous tissue; and layer-by-layer closure of all trocar incisions according to the same standard. A drainage tube was placed in the right subcostal region at the midclavicular line postoperatively.

### 2.6. Outcomes

#### 2.6.1. Primary outcomes.

The primary outcomes included the postoperative VAS scores at extubation and after 30 min, 6 h, 12 h, and 24 h during rest and movement, and the number of PCA uses 24-h postoperatively.

#### 2.6.2. Secondary outcomes.

The secondary outcomes included the following: (1) dermatomal coverage of EOIPB; (2) intraoperative vital signs, including MAP and HR at entry (T0), immediate induction (T1), skin incision (T2), 10 min after the procedure (T3), end of the procedure (T4), and 30 min after extubation (T5); (3) intraoperative dosage of remifentanil; (4) surgical status, including time to first flatus, bed rest time, and hospital stay time; and (5) postoperative recovery quality, with the quality of recovery-40 (QoR-40) [8] of the two groups evaluated 24-h postoperatively. The QoR-40 encompassed five dimensions: self-care ability (5 items), pain sensations (7 items), emotional state (9 items), psychological support (7 items), and physical comfort (12 items), for a total of 40 items, with each item assigned 1–5 points. The total score ranged from 40–200 points. The higher the score was, the better the recovery quality was.

### 2.7. Sample size

Based on data from previous studies, the equivalence margins were set to Δ_1_ = 0.5 for postoperative VAS and Δ_2_ = 1 for PCA boluses. The required sample size was calculated using an equivalence test formula, considering a significance level of α = 0.05, study power of 1 − β = 0.80, the expected standard deviation (σ), and the mean difference between the two groups. After accounting for an anticipated dropout rate of approximately 10%, the final sample size was ultimately determined to be 60 patients.

### 2.8. Statistical analysis

Data were analyzed using SPSS 25 (Armonk, NY: IBM Corp) and R programming language (V4.3.2; R Core Team, Vienna, Austria). Normality was assessed using the Shapiro–Wilk test. Normally distributed data (mean ± standard deviation [SD]) were compared using t-tests, while non-normally distributed data (median [interquartile range]) were analyzed using Mann–Whitney U tests. Categorical data (n, %) were compared with Chi-square or Fisher’s exact tests, where appropriate. Key analyses included two-way repeated-measures mixed-effects ANOVA for VAS scores and MAP, and Heart Rate (HR) over time, Cox proportional hazards regression for time to first flatus, Negative Binomial Regression for Patient-Controlled Analgesia (PCA) presses, and Multivariate Analysis of Variance (MANOVA) for Quality of Recovery-40 (QoR-40) subscores. A *p*-value <0.05 was considered statistically significant. When significant effects were found in ANOVA or MANOVA, post-hoc tests were conducted with Bonferroni correction for multiple comparisons. R was used for analyzing the mixed-effects ANOVA, Cox regression, negative binomial regression, and MANOVA.

## 3. Results

### 3.1. Demographic profile comparison between the two groups

There was no significant difference in terms of age, gender distribution, body mass index (BMI), ASA classification, location of intrahepatic stones (IHSs), or operation time (*p* > 0.05) (Table 1).

**Table 1:** Demographic profile comparison (mean ± SD).

	EOIPB group (n = 30)	Control group (n = 30)	*t*/χ^2^	*p*
Age (years)	51.27 ± 13.35	51.90 ± 9.52	−0.212	0.833
Gender (M/F)	16/14	13/17	0.601	0.438
BMI (kg/m^2^)	24.95 ± 3.3	24.89 ± 4.0	0.063	0.950
ASA grade				
I/II	8/22	12/18	1.200	0.273
IHSs				
Right/left	18/12	20/10	0.287	0.592
Underlying diseases			0.691	0.875
Acute cholecystitis	15	13		
Acute cholangitis	6	7		
Biliary pancreatitis	1	2		
Cholelithiasis	9	11		
Operation time (min)	82.40 ± 17.93	79.27 ± 19.88	0.641	0.542

### 3.2. Comparison of primary outcomes and secondary outcomes: VAS scores at rest and movement

Patients in the EOIPB group reported significantly lower VAS scores both at rest (mean 1.96, 95% CI: 1.83–2.09) and during movement (mean 2.90, 95% CI: 2.73–3.07) compared to the Control group (rest: 2.62, 95% CI: 2.49–2.75; movement: 3.48, 95% CI: 3.32–3.65; *p* < 0.0001 for group effect). A significant group × time interaction was observed for rest pain (*p* < 0.001), but not for movement (*p* = 0.11), indicating differing pain trajectories between groups only at rest (Table 2).

**Table 2:** Comparison of the VAS score at rest with the VAS score at movement over time.

	Group	Mean (95% CI)	Group *p*-value	Time *p*-value	Time:Group
VAS at rest	EOIPB	1.96 (1.83–2.09)	< 0.0001	< 0.0001	< 0.001
	Control	2.62 (2.49–2.75)			
VAS at movement	EOIPB	3.48 (3.32–3.65)	< 0.0001	< 0.0001	0.11
	Control	2.90 (2.73–3.07)			

### 3.3. Comparison of primary outcomes and secondary outcomes

VAS scores at rest and during movement in the EOIPB group were significantly lower than those of the control group 30 min, 6 h, and 12 h after extubation (*p* < 0.05). There was no significant difference in VAS scores at rest or movement between the two groups at 24 h (*p* > 0.05). The number of PCA uses 24-h postoperatively in the EOIPB group (1.60 ± 0.89) was significantly lower than in the control group (2.50 ± 1.11) (*p* < 0.05). In the EOIPB group, the dosage of intraoperative remifentanil (0.84 ± 0.13 mg) was significantly lower than in the control group (0.94 ± 0.14 mg) (*p* < 0.05). The bedridden time in the EOIPB group (23.33 ± 2.81 h) was significantly shorter than in the control group (25.23 ± 3.64 h) (*p* < 0.05), and there was no significant difference in the time to first flatus or hospitalization time between the two groups (*p* > 0.05). The EOIPB group had significantly higher QoR-40 total scores (170.6 ± 5.2) than the control group (165.5 ± 6.3) (*p* < 0.05). There was no significant difference in self-care ability, emotional state, pain sensations, physical comfort, or psychological support between the two groups (*p* > 0.05) (Table 3).

**Table 3:** Comparison of primary outcomes and secondary outcomes (mean ± SD).

	EOIPB group (n = 30)	Control group (n = 30)	*t*	*p*
Primary outcomes				
VAS at rest				
30 min	1.67 ± 0.61	2.93 ± 0.83	−6.761	0.000
6 h	2.17 ± 0.83	2.90 ± 0.40	−4.338	0.000
12 h	2.33 ± 0.80	2.87 ± 0.94	−2.368	0.021
24 h	1.67 ± 0.71	1.77 ± 0.50	−0.628	0.532
VAS at movement				
30 min	2.80 ± 0.66	3.37 ± 0.93	−2.720	0.009
6 h	3.10 ± 0.76	4.07 ± 0.94	−4.370	0.000
12 h	3.30 ± 0.92	3.90 ± 0.96	−2.478	0.016
24 h	2.40 ± 0.93	2.60 ± 0.72	−0.928	0.357
PCA (uses)	1.60 ± 0.89	2.50 ± 1.11	−3.465	0.001
Secondary outcomes				
Remifentanil (mg)	0.84 ± 0.13	0.94 ± 0.14	−2.406	0.019
Time to first flatus (h)	14.50 ± 4.30	15.33 ± 4.91	−0.700	0.487
Bed rest time (h)	23.33 ± 2.81	25.23 ± 3.64	−2.265	0.027
Hospitalization (days)	8.17 ± 1.60	7.93 ± 1.66	0.555	0.581
QoR-40				
Self-care ability	20.5 ± 2.0	20.2 ± 1.9	0.522	0.604
Pain sensations	29.0 ± 2.9	27.6 ± 2.7	1.896	0.063
Emotional state	39.9 ± 1.6	39.0 ± 2.3	1.793	0.078
Psychological support	30.4 ± 1.7	29.5 ± 2.4	1.746	0.086
Physical comfort	50.5 ± 3.2	49.3 ± 2.4	1.644	0.106
Total	170.6 ± 5.2	165.5 ± 6.3	3.405	0.001

### 3.4. Comparison of primary outcomes and secondary outcomes: Dermatomal coverage of EOIPB

**Fig 4:**
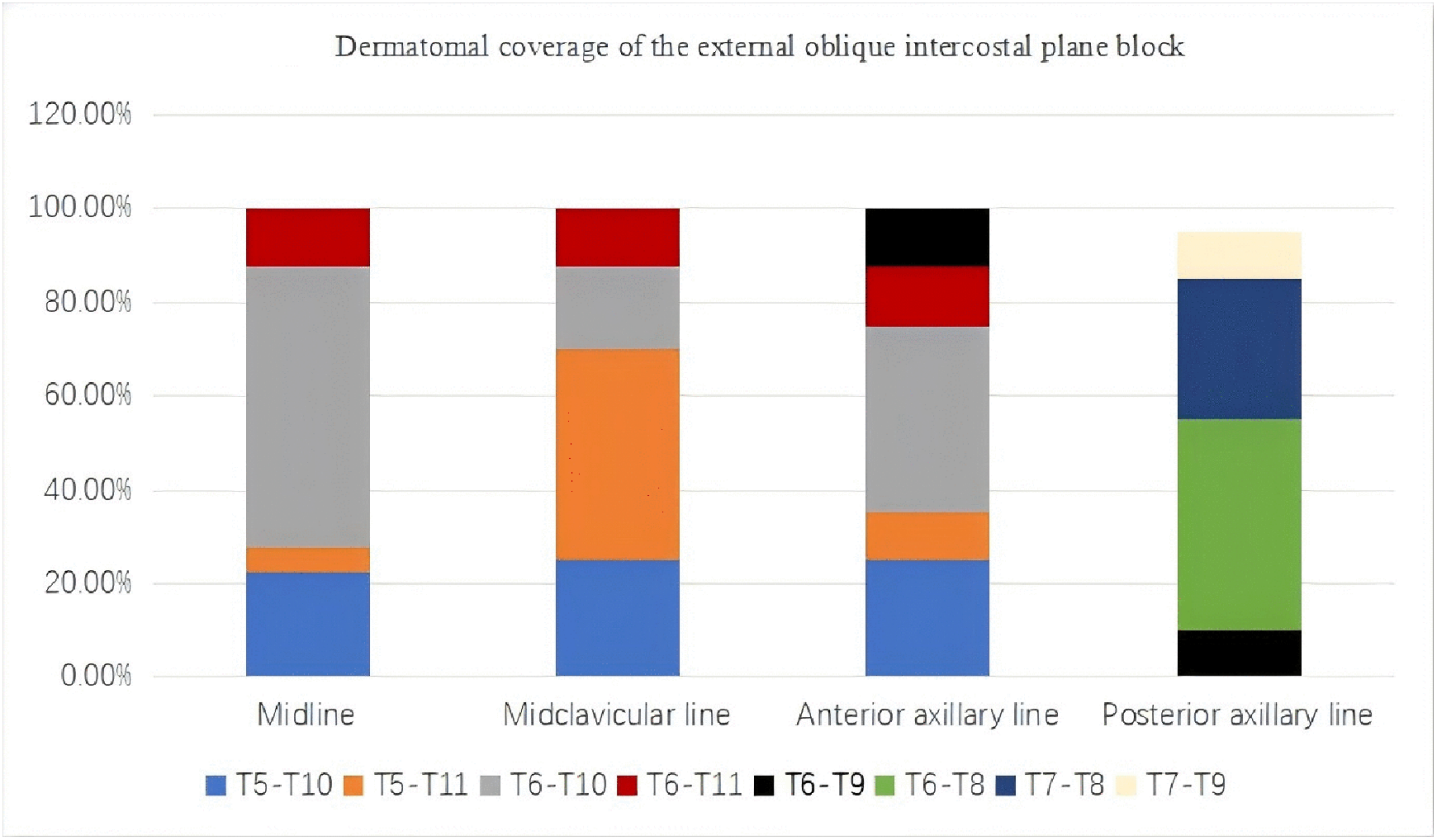
The EOIPB group dermatomal coverage. Of the 30 patients, 28/30 (93.3%) were blocked from the anterior median line to the posterior axillary line. In the anterior median line, 18/30 (60%) had T6–T10 coverage, 6/30 (20%) had T5–T10 coverage, 4/30 (13.3%) had T6–T11 coverage, and 2/30 (0.7%) had T5–T11 coverage. In the midclavicular line, 12/30 (40%) had T5–T11 coverage, 8/30 (26.7%) had T5–T10 coverage, and 5/30 (16.7%) had T6–T10 or T6–T11 coverage. In the anterior axillary line, 12/30 (40%) had T6–T10 coverage, 8/30 (26.7%) had T5–T10 coverage, 4/30 (13.3%) had T6–T11 coverage, and 3/30 (10%) had T5–T11 or T6–T9 coverage. In the axillary posterior line, 14/30 (46.7%) had T6–T8 coverage, 9/30 (30%) had T7–T8 coverage, 4/30 (13.3%) had T7–T9 coverage, and 1/30 (3.3%) had T6–T9 coverage. In the posterior axillary line, 2/30 (6.7%) were not blocked.

In the EOIPB group, the dermatomal coverage achieved using EOIPB was 100% for T6–T10 in the anterior median line and midclavicular line, 100% for T6–T9 in the anterior axillary line, and 93.3% for T7–T8 in the posterior axillary line (Fig 4).

### 3.5. Comparison of intraoperative vital signs

**Fig 5:**
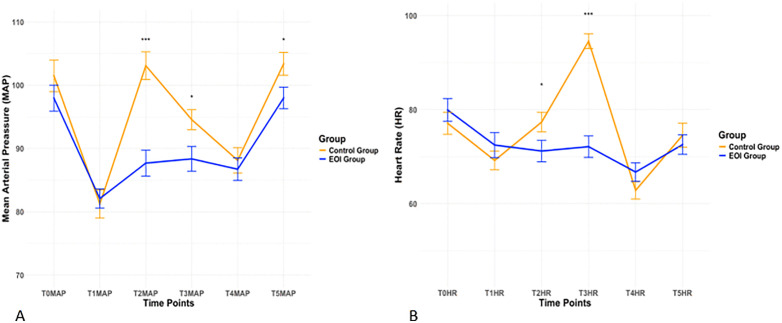
MAP (A) and HR (B) at entry (T0), immediate induction (T1), skin incision (T2), 10 min after the operation (T3), end of the operation (T4), and 30 min after extubation (T5). *** *p* < 0.001 and * *p* < 0.05.

The EOIPB group MAP was significantly lower than the control group MAP at T2, T3, and T5 (*p* < 0.05). The EOIPB group HR was significantly lower than the control group HR at T2 (*p* < 0.05). There was no significant difference in the MAP or HR at other time points (*p* > 0.05, Fig 5A and B).

A two-way repeated-measures mixed-effects ANOVA showed a significant main effect of group (*p* = 0.002), time (*p* < 0.001), and a group × time interaction (*p* < 0.001) for MAP, with post-hoc analysis revealing significantly lower MAP in the EOIPB group at T2, T3, and T5. For HR, there was no significant overall group effect (*p* = 0.092), but time and group × time interaction effects were significant (*p* < 0.001). Post-hoc comparisons showed a significantly lower HR in the EOIPB group at T3 only (*p* < 0.0001) (Table 4).

**Table 4:** Summary of two-way repeated-measures mixed-effect ANOVA analysis and post-hoc test for MAP and HR variables.

Variable	Time	EOIPB Mean (95% CI)	Control Mean (95% CI)	*p*-values		
				Group *p*-value	Time *p*-value	Time:Group *p*-value
MAP	Overall	90.1 (87.9–92.4)	95.3 (93.1–97.6)	0.002	< 0.001	< 0.001
	T0	98.0 (93.4–102.5)	101.5 (96.9–106.1)	0.2796		
	T1	82.1 (78.3–85.9)	81.2 (77.4–85)	0.7488		
	T2	87.7 (83.5–91.9)	103.1 (98.9–107.3)	< 0.0001		
	T3	88.4 (84.8–91.9)	94.6 (91.0–98.1)	0.0164		
	T4	86.7 (82.9–90.5)	88.1 (84.3–91.9)	0.6032		
	T5	98.0 (94.5–101.5)	103.4 (99.9–106.9)	0.0340		
HR	Overall	72.5 (69.7–75.3)	75.9 (73.1–78.8)	0.092	< 0.001	< 0.001
	T0	79.9 (75.2–84.7)	77.1 (72.3–81.9)	0.4048		
	T1	72.5 (67.8–77.2)	69.2 (64.5–73.9)	0.3308		
	T2	71.2 (66.8–75.6)	77.4 (73.0–81.7)	0.0508		
	T3	72.1 (68.2–76.1)	94.6 (90.6–98.5)	< 0.0001		
	T4	66.7 (62.9–70.6)	62.8 (59.0–66.7)	0.1535		
	T5	72.6 (67.9–77.2)	74.6 (69.9–79.2)	0.5462		

### 3.6. Analysis for the Time to First Flatus

**Fig 6:**
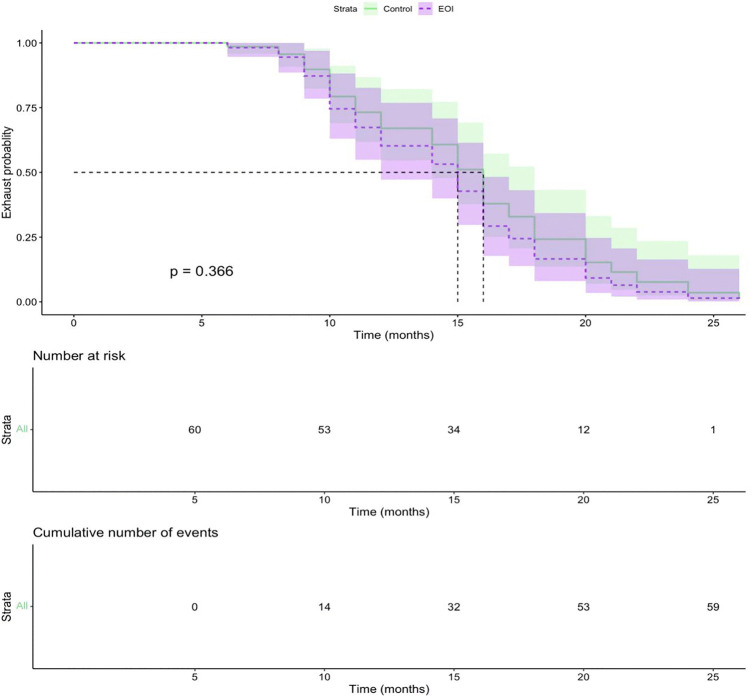
Adjusted Cox proportional hazards model comparing time to first postoperative flatus between the EOIPB and Control groups. The survival curves demonstrate no significant difference between groups after adjusting for age and BMI.

A Cox proportional hazards regression was performed to assess the association between group allocation (EOIPB vs. Control) and the time to first postoperative flatus, adjusting for age and BMI (Fig 6).

The overall model was not statistically significant (Likelihood ratio test: χ^2^(3) = 1.35, *p* = 0.72), indicating no evidence that the covariates jointly influenced time to flatus.

Specifically, the hazard ratio (HR) for the EOIPB group compared to the Control group was 1.27 (95% CI not shown, *p* = 0.366), suggesting a non-significant 27% increase in the rate of instantaneous flatus, adjusting for age and BMI. Age (*p* = 0.800) and BMI (*p* = 0.449) were also not significant predictors.

### 3.7. Negative Binomial Regression Results for PCA Presses (24 h)

The analysis showed that participants in the EOIPB group had significantly fewer PCA presses compared to the Control group (estimate = −0.497, *p* = 0.008). Age and BMI were not significant predictors of PCA presses. There was a trend toward significance for the intercept (*p* = 0.086). Overall, group status was the only significant factor influencing PCA use in 24 h.

### 3.8. EOIPB-related Complications

No EOIPB-related complications (bleeding, infection, hematoma, pneumothorax, or local anesthetic toxicity) were observed in the EOIPB group.

## 4. Discussion

Cholelithiasis is characterized by the presence of gallstones in the liver and bile ducts, and is a common disease in Asia, particularly in East and Southeast Asia [9]. Surgery is the primary treatment for cholelithiasis, and LCBDE has been widely adopted [10,11]. Although LCBDE avoids major trauma and reduces the number of surgical steps, patients may experience severe pain due to the abundance of nerve endings in the surgical area [12]. In this study, all patients successfully completed the surgery. There are three primary sources of pain in LCBDE: the incision site (50%–70% patients), the local and systemic effects of pneumoperitoneum (20%–30% patients), and the liver wound post-operation (10%–20% patients) [13]. Postoperative pain torments the patients physically and mentally, increases the risk of adverse reactions, and delays recovery [14]. Additionally, if patients do not complete recovery protocols, they are prone to postoperative adverse reactions, and therefore, there is a demand for high levels of anesthesia and analgesia [15].

Recently, ultrasound-guided fascial plane block, in which local anesthetics are injected into the fascial planes to provide analgesia for various anatomical regions, has emerged as an alternative to neuraxial techniques. It offers advantages such as longer needle spacing, a lower motor blockade, and fewer hemodynamic changes [16,17]. The innervation of the upper abdominal wall originates from the T6 to T10 intercostal nerves, and effective blockade of these nerves is essential for analgesia [17]. Although there are numerous fascial plane blocks, the subcostal transversus abdominis plane (TAP) block is only effective for upper abdominal wall analgesia and may be ineffective for surgeries near the blocked area, such as the liver and gallbladder [18]. Recently, EOIPB has been described as a novel block for upper abdominal surgeries [6,17]. Hamilton and Manickam proposed that blocking the thoracic fascial plane could block the lateral cutaneous branches of the T7–T11 spinal nerves [19]. In 2019, Hamilton et al. performed EOIPB to provide analgesia for the upper and lateral abdominal walls [20]. White and Ji applied EOIPB on two patients undergoing upper abdominal surgery with an anterior-lateral T7–T11 block plane [21]. In another study, 22 patients received EOIPB, with coverage of the T6–T10 dermatomes at the midclavicular line and T6–T9 at the anterior midline [6]. The surgical incisions for LCBDE are located at the subxiphoid area, the right subcostal region at the midclavicular line, the umbilicus, and the right inferolateral costal region within the T6–T10 dermatomal range. EOIPB is easy to perform, reliable, with minimal side effects, and can be performed in the supine position without requiring special positioning [22,23]. Therefore, EOIPB was selected for our study.

Preemptive analgesia prevents central sensitization by blocking the transmission of nociceptive signals to the spinal cord during surgery, thereby reducing the intensity of pain. It can also diminish the need for pain relief even after the analgesic effects of other potent medications have diminished [24]. The use of fascial plane blocks can reduce the dosage of postoperative analgesics [25,26]. However, no controlled study has reported the intraoperative opioid consumption during the use of EOIPB. Recent controlled studies have shown that EOIPB for upper abdominal surgery was associated with reduced postoperative analgesic use within 24 h and lower VAS scores [27,28]. However, in these studies, EOIPB was performed after anesthesia induction, making it difficult to definitively determine the block’s effectiveness, which may introduce some bias into the conclusions. In this study, EOIPB was performed before anesthesia induction, with dermatomal coverage spanning T6–T10 at the anterior midline and midclavicular line, T6–T9 at the anterior axillary line, and T7–T8 at the posterior axillary line. EOIPB blocks the somatic sensory nerves of the thoracic and abdominal walls. Therefore, its primary analgesic effect is directed toward somatic pain (e.g., incisional pain and referred thoracoabdominal pain). This ensured the effectiveness of the block and generated a preemptive analgesic effect. Additionally, EOIPB can alleviate somatic pain caused by trocar entry incisions. Therefore, in this study, the intraoperative remifentanil dose in the EOIPB group was reduced, and the MAP at T2, T3, T5, and T6 was significantly lower than the corresponding MAP of the control group. Compared to the control group, the HR at T3 and T5 was also lower in the EOIPB group, but the difference was not statistically significant, possibly due to the small sample size. In this study, the EOIPB group had reduced PCA usage within 24 h postoperatively, lower postoperative VAS scores, and shorter bed rest times. This may be because EOIPB blocked nerve fibers related to somatic pain and indirect visceral pain, and also exerted a preemptive analgesic effect. EOIPB does not directly block visceral pain (e.g., pain originating from liver injury or pneumoperitoneum stimulation). However, the following indirect mechanisms may be involved: (1) By effectively alleviating somatic pain, EOIPB can reduce the stress response triggered by somatic stimuli, thereby indirectly attenuating the perception of visceral pain. (2) Reduced intraoperative consumption of remifentanil may lessen the masking effect of opioids on visceral pain, while decreasing opioid-related adverse effects, such as nausea and vomiting, thereby indirectly improving postoperative comfort. EOIPB may cause complications such as vascular injury, hematoma, local anesthetic toxicity, and pneumothorax [29]. In this study, no block-related complications occurred, and the higher QoR-40 scores at 24 h postoperatively indicated that EOIPB accelerated recovery after LCBDE and can be safely and effectively applied in clinical practice.

## 5. Limitations

Preoperative pain scores and symptom burden were not recorded because their potential impact on baseline comparability between groups was not considered in the study design. This may limit the ability to establish baseline comparability between groups. Because our power analysis and sample size determination were optimized for the primary endpoints, findings related to the secondary outcomes, particularly regarding the magnitude of the effects or the interpretation of any non-significant results, should be interpreted with caution. The findings are hypothesis-generating and require validation in larger, multicenter trials. To aid a more reliable interpretation, we have reported effect sizes for these outcomes where appropriate.In addition, the recommended dose, concentration, and volume of local anesthetic for EOIPB have not been determined, which may lead to deviations in the dermatomal coverage of EOIPB and maintenance time. Therefore, more research is needed in the future to determine the optimal local anesthetic dosage, concentration, and volume of EOIPB.

## 6. Conclusions

The application of EOIPB in LCBDE can reduce VAS scores and the number of PCA uses 24 h postoperatively. Furthermore, the use of EOIPB steadied intraoperative vital signs, reduced the remifentanil dose, and improved recovery quality. EOIPB can be safely and effectively used in LCBDE.

## Supporting information

S1 File CONSORT-2010-Checklist. (DOC)

## Abbreviations

EOIPB: external oblique intercostal plane block
LCBDE: laparoscopic common bile duct exploration
PCA: patient-controlled analgesia
QoR-40: quality of recovery score
VAS: visual analog scale
HIS: location of intrahepatic stone

## References

[1] Yang Y, Qiu J, Zhu L. Analysis of EBN interventions in the perioperative period of minimally invasive surgery for intrahepatic and extrahepatic bile duct stones. Altern Ther Health Med. 2022;28(7):139–45. 35839109

[2] Li KY, Shi CX, Tang KL, Huang JZ, Zhang DL. Advantages of laparoscopic common bile duct exploration in common bile duct stones. Wien Klin Wochenschr. 2018;130(3–4):100–4. doi: 10.1007/s00508-017-1232-9 28762058

[3] Zhang W-L, Fang Z-P, Shi B-Y, Chen T, Lv S-D, et al. Low-pressure pulse flushing choledochectomy combined with neodymium laser lithotripsy for the treatment of intrahepatic bile duct stones. Hepatobiliary Pancreat Dis. 2021;20(4):383–6. 10.1016/j.hbpd.2021.04.002 33931315

[4] Desai N, El-Boghdadly K, Albrecht E. Epidural vs. transversus abdominis plane block for abdominal surgery - a systematic review, meta-analysis and trial sequential analysis. Anaesthesia. 2021;76(1):101–17. doi: 10.1111/anae.15068 32385856

[5] Salicath JH, Yeoh EC, Bennett MH. Epidural analgesia versus patient-controlled intravenous analgesia for pain following intra-abdominal surgery in adults. Cochrane Database Syst Rev. 2018;30(8):CD010434. doi: 10.1002/14651858.CD010434.pub2 30161292 PMC6513588

[6] Elsharkawy H, Kolli S, Soliman LM, Seif J, Drake RL, Mariano ER, et al. The external oblique intercostal block: Anatomic evaluation and case series. Pain Med. 2021;22(11):2436–42. doi: 10.1093/pm/pnab296 34626112

[7] Liotiri D, Diamantis A, Papapetrou E, Grapsidi V, Sioka E, Stamatiou G, et al. External oblique intercostal (EOI) block for enhanced recovery after liver surgery: A case series. Anaesth Rep. 2023;11(1):e12225. doi: 10.1002/anr3.12225 37124666 PMC10139870

[8] Sveinsdottir H, Borgthorsdottir T, Asgeirsdottir MT, Albertsdottir K, Asmundsdottir LB. Recovery after same-day surgery in patients receiving general anesthesia: A cohort study using the quality of Recovery-40 Questionnaire. J Perianesth Nurs. 2016;31(6):475–84. doi: 10.1016/j.jopan.2015.07.003 27931699

[9] Liu B, Ma J, Li S, Li C, Qi H, Nian D, et al. Percutaneous transhepatic papillary balloon dilation versus endoscopic retrograde cholangiopancreatography for common bile duct stones: A multicenter prospective study. Radiology. 2021;300(2):470–8. doi: 10.1148/radiol.2021201115 34060938

[10] Ho CT, Le TH, Le VT, Vu VQ, Nguyen HNA, Tran MT. Laparoscopic-cholangioscopic cooperative modified tunnel technique for hepatolithiasis combined with dilated common bile duct: A case report and literature review. Int J Surg Case Rep. 2024;116:109369. doi: 10.1016/j.ijscr.2024.109369 38354574 PMC10943641

[11] Zhang W, Xu G, Huang Q, Luo K, Dong Z, Li J, et al. Treatment of gallbladder stone with common bile duct stones in the laparoscopic era. BMC Surg. 2015;15:7. doi: 10.1186/1471-2482-15-7 25623774 PMC4417333

[12] Yang W, Hu W-L. Effects of intravenously infused lidocaine on analgesia and gastrointestinal function of patients receiving laparoscopic common bile duct exploration. Pak J Med Sci. 2015;31(5):1073–7. doi: 10.12669/pjms.315.7996 26648989 PMC4641258

[13] Mitra S, Khandelwal P, Roberts K, Kumar S, Vadivelu N. Pain relief in laparoscopic cholecystectomy--A review of the current options. Pain Pract. 2012;12(6):485–96. doi: 10.1111/j.1533-2500.2011.00513.x 22008277

[14] Hui V, Hyman N, Viscomi C, Osler T. Implementing a fast-track protocol for patients undergoing bowel resection: Not so fast. Am J Surg. 2013;206(2):152–8. doi: 10.1016/j.amjsurg.2012.11.019 23759698

[15] Uzun S, Erden IA, Canbay O, Aypar U. The effect of intravenous paracetamol for the prevention of rocuronium injection pain. Kaohsiung J Med Sci. 2014;30(11):566–9. doi: 10.1016/j.kjms.2014.08.002 25458046 PMC11916363

[16] Macrae WA. Chronic post-surgical pain: 10 years on. Br J Anaesth. 2008;101(1):77–86. doi: 10.1093/bja/aen099 18434337

[17] Elsharkawy H, Pawa A, Mariano ER. Interfascial plane blocks: Back to basics. Reg Anesth Pain Med. 2018;43(4):341–6. doi: 10.1097/AAP.0000000000000750 29561295

[18] Ozciftci S, Sahiner Y, Sahiner IT, Akkaya T. Is Right Unilateral Transversus Abdominis Plane (TAP) block successful in postoperative analgesia in laparoscopic cholecystectomy?. Int J Clin Pract. 2022;2022:2668215. doi: 10.1155/2022/2668215 35685608 PMC9159215

[19] Hamilton DL, Manickam BP. Is a thoracic fascial plane block the answer to upper abdominal wall analgesia? Reg Anesth Pain Med 2018 Nov;43(8):891–2. 30339615 10.1097/AAP.0000000000000838

[20] Hamilton DL, Manickam BP, Wilson MAJ, Abdel Meguid E. External oblique fascial plane block. Reg Anesth Pain Med. 2019;:rapm-2018-100256. doi: 10.1136/rapm-2018-100256 30635518

[21] White L, Ji A. External oblique intercostal plane block for upper abdominal surgery: Use in obese patients. Br J Anaesth. 2022;128(5):e295–7. doi: 10.1016/j.bja.2022.02.011 35249704

[22] Tran DQ, Boezaart AP, Neal JM. Fascial plane blocks: The next leap. Reg Anesth Pain Med. 2021;46(7):568–9. doi: 10.1136/rapm-2020-101992 34145068

[23] Hotta K. Fascial plane blocks: Anatomical structures that affect the spread of local anesthetic. J Anesth. 2019;33(4):493–4. doi: 10.1007/s00540-019-02647-z 31073655

[24] Katz J. Pre-emptive analgesia: Importance of timing. Can J Anaesth. 2001;48(2):105–14. doi: 10.1007/BF03019721 11220417

[25] Ragupathy R, Prabhu SCG, Thiyagarajan D, Anto V. Opioid-free anaesthesia for laparoscopic surgeries - A prospective non-randomised study in a tertiary care hospital. Indian J Anaesth. 2022;66(3):207–12. doi: 10.4103/ija.ija_785_21 35497703 PMC9053893

[26] Çömez MS, Sağlambilen H, Çelik ECE, Koyuncu O, Hakimoğlu S, et al. Efficacy of unilateral external oblique intercostal fascial plane block versus subcostal TAP block in laparoscopic cholecystectomy: Randomized, prospective study. Surg Innov. 2024. doi: 10.1177/15533506241256529 38780355 PMC11264529

[27] Korkusuz M, Basaran B, Et T, Bilge A, Yarimoglu R, Yildirim H. Bilateral external oblique intercostal plane block (EOIPB) in patients undergoing laparoscopic cholecystectomy: A randomized controlled trial. Saudi Med J. 2023;44(10):1037–46. doi: 10.15537/smj.2023.44.10.20230350 37777270 PMC10541983

[28] Kavakli AS, Sahin T, Koc U, Karaveli A. Ultrasound-guided external oblique intercostal plane block for postoperative analgesia in laparoscopic sleeve gastrectomy: A prospective, randomized, controlled, patient and observer-blinded study. Obesity Surgery. 2024;34(5):1505–12. doi: 10.1007/s11695-024-07174-9 38499943 PMC11031435

[29] Erskine RN, White L. “A review of the external oblique intercostal plane block - a novel approach to analgesia for upper abdominal surgery”. J Clin Anesth. 2022;82:110953. doi: 10.1016/j.jclinane.2022.110953 35994942

