# Supplementary material for: Efficacy of ultrasound-guided external oblique intercostal plane block in laparoscopic common bile duct exploration: A double-blinded randomized controlled study
Source: PLoS One. 2026 Aug 6;21(8):e0355377. doi: 10.1371/journal.pone.0355377 (PMC13446742; doi:10.1371/journal.pone.0355377)
Supplement: Supplementary file 4 [file pone.0355377.s003.doc]

April 29, 2026

Chong-Chi Chiu

Academic Editor, *PLOS ONE*

Response to Editor:

Dear Dr. Chong-Chi Chiu,

We sincerely appreciate the reviewers’ thorough and constructive comments, which have substantially improved the rigor, transparency, and reporting quality of our manuscript. We have addressed each comment point-by-point and revised the manuscript accordingly, with all changes clearly indicated in the revised manuscript.

In particular, we have fully addressed all reviewer comments, enhanced methodological transparency, strengthened reporting in accordance with the **CONSORT 2010 guidelines**, and clarified limitations related to sample interpretation. We believe the revised manuscript is now suitable for publication in PLOS ONE.

Thank you again for your valuable time and consideration. We look forward to your response.

Yours sincerely,

Qianqian Jia

Address: Wenhua Road 258, Qinhuangdao, China.

Email: jiaqian040@126.com

## Response to Reviewer 1

**Reviewer #1: The analysis is much improved. The sample size issue was not addressed. It should at lease be presented as a convenience sample which cautions the interpretation of the p-values.**

**Response:** We sincerely acknowledge this important comment. We have fully revised the **Sample Size** and **Limitations** sections.

We have supplemented the complete sample size calculation process based on an equivalence framework, including the predefined equivalence margins (Δ_1_ = 0.5 for VAS and Δ_2_ = 1 for PCA boluses), a two-sided significance level of α = 0.05, study power of 0.80, the standard deviation (SD) from pilot data, and an anticipated 10% dropout rate. This resulted in a target sample size of 60 patients (Page 7, Lines 193–199).

Although the sample size was calculated a priori, we acknowledge that the final sample.Remains relatively small and was derived from a single center, which limits generalizability. We have explicitly stated in the **Limitations** section that this study should be considered a **convenience sample** and that statistical significance and p-values should be interpreted with caution. The findings are hypothesis-generating and require validation in larger, multicenter trials (Page 16, Lines 395–399).

## Response to Reviewer 2

**Reviewer #2: While this topic is potentially interesting, I have several concerns about the study design:**

1. **I do not think this clinical trial could be double-blind.**

**Response:** Thank you for this important reminder. We agree that **full double-blinding is not feasible** because EOIPB is an invasive regional block procedure; therefore, both patients and the anesthesiologist performing the block cannot be blinded to group allocation. We have clarified in the revised manuscript that this trial used **assessor–statistician blinding**:

Patients and the anesthesiologist performing EOIPB were aware of group assignment. Outcome assessors (VAS recording), anesthesiologists responsible for general anesthesia, and statisticians were **fully blinded** to group allocation throughout the study. This blinding approach is clearly described in the Methods section to avoid misinterpretation (Page 3, Lines 87–100).

2. Please state how to randomly assign the patient.

**Response:** Randomization was performed using a **random number table** prior to study initiation.

Eligible patients were randomized in a 1:1 ratio to the EOIPB group or the control group using **sealed, sequentially numbered opaque envelopes**. A research assistant (not involved in the study intervention, anesthesia management, or data collection) opened the envelopes and assigned patients to groups. The allocation was disclosed only to the physician responsible for performing EOIPB, while the anesthesiologists, **statisticians**, and observers remained blinded to group assignment. To ensure, allocation concealment, the observer remained outside the operating room during block performance and anesthesia induction. Full details have now been included in the Study Design section (Page 2, Lines 87–94).

**3. Please add the inclusion and exclusion criteria in the method section, and please add more detail process about how to enroll study subjects.**

**Response:** We have added the complete **inclusion and exclusion criteria, as well as recruitment procedure,** to the Study Design section:

Inclusion criteria were as follows: aged 18–65 years; American Society of Anesthesiologists (ASA) physical status I–II; diagnosis of choledocholithiasis and scheduled for elective laparoscopic common bile duct exploration (LCBDE); clear consciousness; ability to cooperate with postoperative pain scoring and follow-up; and provision of written informed consent. Exclusion criteria were as follows: history of liver disease, alcoholism, or drug abuse; mental illness; prior abdominal surgery or trauma; long-term opioid use; coagulation disorders; systemic or local infection at the injection site; conversion to open surgery during the procedure, or allergy to local anesthetics (Page 3, Lines 78–86).

### **Recruitment Procedure**

Patients were screened preoperatively by a dedicated anesthesiologist. Those meeting the eligibility criteria were provided with a detailed explanation of the study purpose, procedures, and potential risks and benefits. After providing written informed consent, eligible patients were enrolled and randomized as described above. During the preoperative visit, baseline assessments and VAS/PCA training were conducted by the observer (Page 2, Lines 87–99).

## Response to Reviewer 3

### Reviewer #3: This manuscript reports a randomized controlled trial evaluating the efficacy of ultrasound-guided external oblique intercostal plane block (EOIPB) specifically in laparoscopic common bile duct exploration (LCBDE).

### The reviewer has inquiries regarding the study design, and the selection of LCBDE as the procedure of choice for the trial:

1. The manuscript mentioned that a left-sided EOIPB was performed. What was the rationale for performing the block on the left side, when the target anatomy and incisions for LCBDE are typically on the right side?

**Response:** Thank you for your reminder. We sincerely apologize for this error. As shown in Figure 1, the procedure performed was a right-sided EOIPB. We have corrected this in the revised manuscript and uploaded the revised manuscript (Page 1, Line 22; Page 4, Line 117).

2. **LCBDE is often considered an adjunct to laparoscopic cholecystectomy, when indicated, and typically does not involved any additional incisions. Why was LCBDE selected for the study, and not the more prevalent/common cholecystectomy procedure. The Discussion section briefly mentions the locations of the incisions made for the procedure. Can the authors provide details on the size of the incisions, and whether fascial closure was performed at each of these incisions at the conclusion of the procedure, as the fascial closure can often be a significant contributor to postoperative pain.**

****Response:****

LCBDE was selected because it involves **greater visceral traction, longer operative time, drainage tube insertion, and more intense postoperative pain** (including somatic pain, visceral pain, and drainage tube-related irritation) compared with standard cholecystectomy. This model allows for better detection of analgesic effects of EOIPB.

The trocar sites and sizes were as follows: umbilicus (1.0–1.2 cm); subxiphoid region (0.5 cm); right subcostal region at the midclavicular line (1.5–2.0 cm); and right anterior axillary line (drain site, 0.5 cm).

**Fascial closure was performed for all incisions** at the end of surgery using standardized suturing techniques by surgeons from the same surgical team. These details have now been included to the Surgical Procedure section (Pages 6, Lines 156–171).

3. **Was the effects of the EOIPB limited to somatic pain, or were there effects on visceral pain as well?**

**Response:** Thank you for pointing this out. EOIPB mainly blocks **somatic pain** (skin, muscle, and parietal peritoneum) by interrupting this intercostal nerves and has **no direct effect on visceral pain**. However, it may indirectly improve overall pain perception through several mechanisms: reduced abdominal wall tenderness and guarding; attenuation of the stress response and secondary hyperalgesia; and decreased opioid consumption and related adverse effects. This distinction has been clarified in the Discussion (Page 15, Lines 371–373; Page 16, Lines 381–389).

**4. The authors also mentioned that the effects of pneumoperitoneum can contribute to postoperative pain. What was the degree of pneumoperitoneum used for the LCBDE procedures, and what was the average operative time?**

**Response:**

Intraoperative CO_2_ pneumoperitoneum was established at an insufflation rate of 2–4 L/min. Intra-abdominal pressure was **maintained at 12**–**14 mmHg** and dynamically adjusted by the anesthesiologist based on the patient's vital signs (blood pressure, heart rate, and airway pressure) and surgical requirements (Page 6, Lines 157–160).

The mean operative time was 82.40±17.93 min in the EOIPB group and 79.27±19.88 min in the control group (*p*>0.05) (Page 8-9, Table 1).

**The reviewer also find that some essential information was missing from the manuscript, and further details on the execution of the trial is needed. Please adhere strictly to the CONSORT reporting guidelines for a complete list of the information needed:**

1. **How were participants identified for initial screening for in the trial? Additionally, more information is needed on how randomization was performed.**

**Response:**

**Initial screening**: A dedicated anesthesiologist reviewed medical records, conducted a preoperative evaluation, assessed inclusion and exclusion criteria, and confirmed patient eligibility (Page 3, Lines 77–88).

**Randomization**: Randomization was performed using a **random number table** before study initiation. Eligible patients were randomized in a 1:1 to the EOIPB group or the control group using **sealed, sequentially numbered opaque envelopes**. A research assistant (not involved in the study intervention, anesthesia management, or data collection) opened the envelopes and assigned patients to groups. The allocation was disclosed only to the physician responsible for EOIPB, while the anesthesiologists, surgeons, and observers remained blinded to group assignment. To ensure allocation concealment, the observer remained outside the operating room during block performance and anesthesia induction. Full details have been included in the Study Design section (Page 3, Lines 87–92).

2. **In this study, it is unclear how the patient can be blinded to the intervention, as the EOIPB is performed prior to anesthesia induction. Similarly, is the interventionalist performing the block separate from the anesthesiologist for the case, to ensure that the anesthesiologist is blinded to the assigned treatment? How did the anesthesiologist determine the dose of remifentanil to the administered intraoperatively, as this measure was an outcome reported for the study?**

**Response:** Thank you for your reminder. We agree that full double-blinding is not feasible because EOIPB is an invasive regional block procedure; therefore, patients and the anesthesiologist performing the block cannot be blinded to group allocation. We have clearly stated in the revised manuscript that this trial employed assessor–statistician blinding:

Patients and the anesthesiologist performing EOIPB were aware of group assignment. Outcome assessors (VAS recording), anesthesiologists responsible for general anesthesia, and statisticians were fully blinded to group allocation throughout the study. This blinding design is clearly described in the Study Design section to avoid misinterpretation (Page 3-4, Lines 87–100).

Remifentanil was administered at a basal infusion rate of 0.1 ug/kg/min. The intraoperative infusion rate was adjusted based on heart rate (HR), mean arterial pressure (MAP), sweating, lacrimation, coughing, and body movement. If pain-related responses occurred, the infusion rate was increased by 0.05 ug/kg/min. If bradycardia or hypotension occurred, the infusion rate was decreased by 0.05 ug/kg/min. The bispectral index (BIS) was maintained between 40 and 60. These details are fully described in the Anesthesia management section (Page 5, Lines 137–141).

3. Postoperatively, was adjunctive pain control solely limited to the PCA? Or were there additional analgesics available as needed?

**Response:** Postoperative analgesia was **primarily maintained with patient-controlled intravenous analgesia (PCIA).** Patients with visual analog scale (VAS) scores ≥4 or those requesting additional analgesia received PCA as rescue analgesia. If analgesic efficacy was suboptimal, PCA administration could be repeated after 15 minutes following physician evaluation to ensure adequate pain control. No additional analgesics were administered within 24 h postoperatively. This has now been clarified in Section 2.4 (Postoperative Analgesia) (Page 5-6, Lines 146–151).

4. The EOIPB was performed in the operating room, prior to the induction of anesthesia. The Methods section also indicates that the block was tested 15 minutes later, again prior to anesthesia induction. Did this intervention lead to delays in the operating room? What was the mean duration from entry into the operating room to skin incision, and was it different between the intervention and control arms? Were there other post-procedural complications related to the EOIPB?

**Response:**

Thank you for pointing this out. We acknowledge that this aspect was not described in sufficient detail in the original manuscript. EOIPB and block assessment were performed **in the pre-anesthesia room** rather than in the operating room; therefore, **no operating room delay occurred**. To ensure adequate block onset, patients in the EOIPB group were transferred to the operating room **15 minutes earlier**, as explained during the informed consent process. All patients were transferred from the pre-anesthesia room to the operating room (Page 4, Lines 101–106).

**No EOIPB-related complications** (bleeding, infection, hematoma, pneumothorax, or local anesthetic toxicity) were observed in any patient. These data have been added to the Results section (Page 14, Lines 322–324).
