# Supplementary material for: Efficacy of ultrasound-guided external oblique intercostal plane block in laparoscopic common bile duct exploration: A double-blinded randomized controlled study
Source: PLoS One. 2026 Aug 6;21(8):e0355377. doi: 10.1371/journal.pone.0355377 (PMC13446742; doi:10.1371/journal.pone.0355377)
Supplement: Supplementary file 2 [file pone.0355377.s004.docx]

June 18, 2026

Chong-Chi Chiu

Academic Editor, *PLOS ONE*

Response to Editor:

Dear Dr. Chiu,

We would like to sincerely thank the Academic Editor and all reviewers for their valuable comments and constructive suggestions on our manuscript. We have carefully considered all feedback and revised the manuscript accordingly. Below, we provide a point-by-point response to the comments from Reviewer 3. All revisions have been highlighted in the revised manuscript using Track Changes.

Once again, we thank the reviewers and Academic Editor for your careful evaluation and insightful recommendations. We believe that the resulting revisions have fstrengthened the completeness and rigor of the study. We hope that the revised manuscript now meets the requirements for publication in PLOS ONE.

Thank you again for your valuable time and consideration. We are looking forward to your positive decision.

Yours sincerely,

Qianqian Jia

Address: Wenhua Road 258, Qinhuangdao, China.

Email: jiaqian040@126.com

Response to Reviewer 3

Comment 1: More detailed demographic and clinical information regarding the patient populations in both study groups would be valuable. Specifically, the authors should report the underlying pathology necessitating laparoscopic common bile duct exploration (LCBDE), including the presence of acute cholecystitis, biliary pancreatitis, cholangitis, or other relevant conditions. Additional information regarding the etiology of common bile duct obstruction (e.g., choledocholithiasis, benign stricture, malignancy, or other causes) would also improve the characterization of the study cohort. Furthermore, reporting preoperative pain severity or symptom burden would help establish baseline comparability between groups.

Response:

Thank you for this important suggestion. We agree that detailed clinical characteristics can better characterize the study population and help verify baseline comparability between groups.

In response to this comment, we have supplemented the relevant information in the Study Design and Results sections of the revised manuscript:

We added a detailed classification of the primary underlying diseases for all enrolled patients, including acute cholecystitis, acute cholangitis, biliary pancreatitis and simple cholelithiasis with choledocholithiasis , and compared their distribution between the two groups (Page 8-9, Table1).

We clarified the etiology of common bile duct obstruction and specified that all included patients were diagnosed with choledocholithiasis (Page 3, Line 79); no patients with benign biliary stricture or malignant biliary obstruction were enrolled in this trial , and this information has now been explicitly stated in the manuscript (Page 3, Lines 82-84).

Due to an oversight in the study design, preoperative pain scores and symptom burden were not recorded, as the potential impact of these variables on the baseline comparability between groups was not considered.We acknowledge that this may limit the ability to establish baseline comparability between groups. This limitation has been added to the Limitations section of the revised manuscript (Page 16, Lines 402-404).

Comment 2: The manuscript would also benefit from greater detail regarding the operative techniques employed during LCBDE. Specifically, the authors should clarify whether stone extraction was performed using basket retrieval, balloon catheter extraction, choledochoscopy-assisted techniques, sphincterotomy, or choledochotomy with direct stone removal. Information regarding bile duct closure methods, if applicable, would also be helpful. Finally, operative duration should be reported and compared between groups, as it represents an important procedural outcome and may influence perioperative results.

Response:

Thank you for this valuable suggestion regarding the reporting of surgical technical details. In response, we have extended the Surgical Procedures section to provide a more detailed and standardized description of the laproscopic common bile duct exploration (LCBDE) procedure.

1) Surgical techniques: We added a detailed description of the LCBDE procedure in the Surgical Procedures section. All patients underwent laparoscopic choledochotomy combined with choledochoscopy-assisted stone extraction. For most calculi, choledochoscope-guided basket retrieval was the preferred method of stone extraction. For small, sandy, or residual calculi, balloon catheter extraction was used as an adjunctive technique to facilitate biliary tract clearance. No endoscopic sphincterotomy was performed in any of the enrolled patients (Page 5-6, Lines 148-158).
2) Bile duct closure: After complete stone clearance, primary closure of the common bile duct was performed in all patients without T-tube drainage. Details regarding the suture material and suturing technique have also been added to the manuscript (Page 5-6, Lines 158-162).
3) Operative duration: We recorded and analyzed the total operative time both groups in the Results section. No significant difference in operative duration was observed between the two groups (*P*>0.05), suggesting that operative time was unlikely to have confounded perioperative pain outcomes or other study endpoints(Page 9, Table 1).
