# Supplementary material for: Efficacy of ultrasound-guided external oblique intercostal plane block in laparoscopic common bile duct exploration: A double-blinded randomized controlled study
Source: PLoS One. 2026 Aug 6;21(8):e0355377. doi: 10.1371/journal.pone.0355377 (PMC13446742; doi:10.1371/journal.pone.0355377)
Supplement: Peer review history

# Peer review: Decision Letter 0

**DOI:** 10.1371/journal.pone.0355377.r001

**Authors:** Mohamed Ali Chaouch

2 May 2025

PONE-D-25-08783

Efficacy of ultrasound-guided external oblique intercostal plane block in laparoscopic common bile duct exploration: a randomized controlled study

PLOS ONE

Dear Dr. Jia,

Thank you for submitting your manuscript to PLOS ONE. After careful consideration, we feel that it has merit but does not fully meet PLOS ONE’s publication criteria as it currently stands. Therefore, we invite you to submit a revised version of the manuscript that addresses the points raised during the review process.

Please submit your revised manuscript by Jun 16 2025 11:59PM. If you will need more time than this to complete your revisions, please reply to this message or contact the journal office at plosone@plos.org. When you're ready to submit your revision, log on to https://www.editorialmanager.com/pone/ and select the 'Submissions Needing Revision' folder to locate your manuscript file.

Please include the following items when submitting your revised manuscript:

- A rebuttal letter that responds to each point raised by the academic editor and reviewer(s). You should upload this letter as a separate file labeled 'Response to Reviewers'.
- A marked-up copy of your manuscript that highlights changes made to the original version. You should upload this as a separate file labeled 'Revised Manuscript with Track Changes'.
- An unmarked version of your revised paper without tracked changes. You should upload this as a separate file labeled 'Manuscript'.

If you would like to make changes to your financial disclosure, please include your updated statement in your cover letter. Guidelines for resubmitting your figure files are available below the reviewer comments at the end of this letter.

If applicable, we recommend that you deposit your laboratory protocols in protocols.io to enhance the reproducibility of your results. Protocols.io assigns your protocol its own identifier (DOI) so that it can be cited independently in the future. For instructions see: https://journals.plos.org/plosone/s/submission-guidelines#loc-laboratory-protocols. Additionally, PLOS ONE offers an option for publishing peer-reviewed Lab Protocol articles, which describe protocols hosted on protocols.io. Read more information on sharing protocols at https://plos.org/protocols?utm_medium=editorial-email&utm_source=authorletters&utm_campaign=protocols.

We look forward to receiving your revised manuscript.

Kind regards,

Mohamed Ali Chaouch

Academic Editor

PLOS ONE

Journal requirements:

When submitting your revision, we need you to address these additional requirements.

1. Please ensure that your manuscript meets PLOS ONE's style requirements, including those for file naming. The PLOS ONE style templates can be found at

https://journals.plos.org/plosone/s/file?id=wjVg/PLOSOne_formatting_sample_main_body.pdf and

https://journals.plos.org/plosone/s/file?id=ba62/PLOSOne_formatting_sample_title_authors_affiliations.pdf.

2. We note that you have selected “Clinical Trial” as your article type. PLOS ONE requires that all clinical trials are registered in an appropriate registry (the WHO list of approved registries is at https://www.who.int/clinical-trials-registry-platform/network/primary-registries" https://www.who.int/clinical-trials-registry-platform/network/primary-registries and more information on trial registration is at http://www.icmje.org/about-icmje/faqs/clinical-trials-registration/). Please state the name of the registry and the registration number (e.g. ISRCTN or ClinicalTrials.gov) in the submission data and on the title page of your manuscript. a) Please provide the complete date range for participant recruitment and follow-up in the methods section of your manuscript. b) If you have not yet registered your trial in an appropriate registry, we now require you to do so and will need confirmation of the trial registry number before we can pass your paper to the next stage of review. Please include in the Methods section of your paper your reasons for not registering this study before enrolment of participants started. Please confirm that all related trials are registered by stating: “The authors confirm that all ongoing and related trials for this drug/intervention are registered”. Please see http://journals.plos.org/plosone/s/submission-guidelines#loc-clinical-trials for our policies on clinical trials.

3.  Thank you for stating the following financial disclosure:

[The study was supported by Science and Technology Program of Qinhuangdao City (Project Number: 202301A269).].

Please state what role the funders took in the study.  If the funders had no role, please state: ""The funders had no role in study design, data collection and analysis, decision to publish, or preparation of the manuscript.""

If this statement is not correct you must amend it as needed.

Please include this amended Role of Funder statement in your cover letter; we will change the online submission form on your behalf.

4. We note that your Data Availability Statement is currently as follows: [All relevant data are within the manuscript and its Supporting Information files.]

Please confirm at this time whether or not your submission contains all raw data required to replicate the results of your study. Authors must share the “minimal data set” for their submission. PLOS defines the minimal data set to consist of the data required to replicate all study findings reported in the article, as well as related metadata and methods (https://journals.plos.org/plosone/s/data-availability#loc-minimal-data-set-definition).

For example, authors should submit the following data:

- The values behind the means, standard deviations and other measures reported;

- The values used to build graphs;

- The points extracted from images for analysis.

Authors do not need to submit their entire data set if only a portion of the data was used in the reported study.

If your submission does not contain these data, please either upload them as Supporting Information files or deposit them to a stable, public repository and provide us with the relevant URLs, DOIs, or accession numbers. For a list of recommended repositories, please see https://journals.plos.org/plosone/s/recommended-repositories.

If there are ethical or legal restrictions on sharing a de-identified data set, please explain them in detail (e.g., data contain potentially sensitive information, data are owned by a third-party organization, etc.) and who has imposed them (e.g., an ethics committee). Please also provide contact information for a data access committee, ethics committee, or other institutional body to which data requests may be sent. If data are owned by a third party, please indicate how others may request data access.

5. When completing the data availability statement of the submission form, you indicated that you will make your data available on acceptance. We strongly recommend all authors decide on a data sharing plan before acceptance, as the process can be lengthy and hold up publication timelines. Please note that, though access restrictions are acceptable now, your entire data will need to be made freely accessible if your manuscript is accepted for publication. This policy applies to all data except where public deposition would breach compliance with the protocol approved by your research ethics board. If you are unable to adhere to our open data policy, please kindly revise your statement to explain your reasoning and we will seek the editor's input on an exemption. Please be assured that, once you have provided your new statement, the assessment of your exemption will not hold up the peer review process.

6.  Your ethics statement should only appear in the Methods section of your manuscript. If your ethics statement is written in any section besides the Methods, please delete it from any other section.

[Note: HTML markup is below. Please do not edit.]

Reviewers' comments:

Reviewer's Responses to Questions

**Comments to the Author**

1. Is the manuscript technically sound, and do the data support the conclusions?

The manuscript must describe a technically sound piece of scientific research with data that supports the conclusions. Experiments must have been conducted rigorously, with appropriate controls, replication, and sample sizes. The conclusions must be drawn appropriately based on the data presented.

Reviewer #1: Partly

**********

2. Has the statistical analysis been performed appropriately and rigorously?

Reviewer #1: No

**********

3. Have the authors made all data underlying the findings in their manuscript fully available?

The PLOS Data policy requires authors to make all data underlying the findings described in their manuscript fully available without restriction, with rare exception (please refer to the Data Availability Statement in the manuscript PDF file). The data should be provided as part of the manuscript or its supporting information, or deposited to a public repository. For example, in addition to summary statistics, the data points behind means, medians and variance measures should be available. If there are restrictions on publicly sharing data—e.g. participant privacy or use of data from a third party—those must be specified.

Reviewer #1: Yes

**********

4. Is the manuscript presented in an intelligible fashion and written in standard English?

PLOS ONE does not copyedit accepted manuscripts, so the language in submitted articles must be clear, correct, and unambiguous. Any typographical or grammatical errors should be corrected at revision, so please note any specific errors here.

Reviewer #1: Yes

**********

5. Review Comments to the Author

Please use the space provided to explain your answers to the questions above. You may also include additional comments for the author, including concerns about dual publication, research ethics, or publication ethics. (Please upload your review as an attachment if it exceeds 20,000 characters)

Reviewer #1: The objective of the study was to observe the efficacy of EOIPB in patients undergoing LCBDE. Sixty patients undergoing elective LCBDE were randomly divided into two groups:

EOI group and control group. The patients in the EOI group received ultrasound-guided EOIPB before anesthesia induction and the patients in the control group received no intervention. It was a very simple parallel design.

The analysis was oversimplified with basically univariate analysis involving t-test for normally distributed variables and the Mann-Whitney U test for non-normally distributed variables. Categorical variables were expressed as the number (n) and percentage (%), with the Chi-square test and Fisher's exact test used for categorical data. The sample size was addressed in the supplemental protocol. The results were expressed accordingly.

There are several issues which , if addressed, could enhance the study.

1. What was the sample size software used?

2. The strategy calls for several assessment time points. The sample size appears to be based on one assessment. This may have to be re-assessed. The design is actually a two way ANOVA of treatment at two levels (EOIPB and control) and time at 5 levels (rest, 30min ,6h,12h, 24h) plus possible interactions as seen by Figures 5 and 6.

The current analysis is not very sophisticated and could be improved according to this design.

3. There are also secondary objectives. How will these assessments affect the sample size?

4. According to section 10 of the protocol and methods section of the manuscript, it seems that other variables will be collected on the subjects. Why is there no multivariate analysis built into the statistical assessment. The design could be an analysis of covariance depending on patient and clinical data (as well as treatment and time) collected influencing the outcomes.

5. The protocol has no section on data management and quality control. How was this performed?

6. Table 2 plus the text presentation presents multiple univariate statistical tests. Was there any adjustment of the type one error to control for these multiple comparisons?

The paper requires a re-assessment of the statistical presentation and analysis.

**********

6. PLOS authors have the option to publish the peer review history of their article (what does this mean?). If published, this will include your full peer review and any attached files.

If you choose “no”, your identity will remain anonymous but your review may still be made public.

**Do you want your identity to be public for this peer review?**  For information about this choice, including consent withdrawal, please see our Privacy Policy.

Reviewer #1: No

**********

[NOTE: If reviewer comments were submitted as an attachment file, they will be attached to this email and accessible via the submission site. Please log into your account, locate the manuscript record, and check for the action link "View Attachments". If this link does not appear, there are no attachment files.]

While revising your submission, please upload your figure files to the Preflight Analysis and Conversion Engine (PACE) digital diagnostic tool, https://pacev2.apexcovantage.com/. PACE helps ensure that figures meet PLOS requirements. To use PACE, you must first register as a user. Registration is free. Then, login and navigate to the UPLOAD tab, where you will find detailed instructions on how to use the tool. If you encounter any issues or have any questions when using PACE, please email PLOS at figures@plos.org. Please note that Supporting Information files do not need this step.

# Author comment: Author response to Decision Letter 1

**DOI:** 10.1371/journal.pone.0355377.r002

12 Jun 2025

1. We note that the grant information you provided in the ‘Funding Information’ and ‘Financial Disclosure’ sections do not match.

When you resubmit, please ensure that you provide the correct grant numbers for the awards you received for your study.

In addition, please state what role the funders took in the study. If the funders had no role, please state: "The funders had no role in study design, data collection and analysis, decision to publish, or preparation of the manuscript."

Kindly include this amended Funding disclosure statement in your cover letter; we will change the online submission form on your behalf.

Response: Thank you for pointing this out. The correct funding information is as followed.

The study was supported by the Science and Technology Program of Qinhuangdao City (Project Number: 202301A269). The funders had no role in study design, data collection and analysis, decision to publish, or preparation of the manuscript. (Page 14-15, Line 339-342)

2. When completing the data availability statement of the submission form, you indicated that you will make your data available on acceptance. We strongly recommend all authors decide on a data sharing plan before acceptance, as the process can be lengthy and hold up publication timelines. Please note that, though access restrictions are acceptable now, your entire data will need to be made freely accessible if your manuscript is accepted for publication. This policy applies to all data except where public deposition would breach compliance with the protocol approved by your research ethics board. If you are unable to adhere to our open data policy, please kindly revise your statement to explain your reasoning and we will seek the editor's input on an exemption. Please be assured that, once you have provided your new statement, the assessment of your exemption will not hold up the peer review process.

Response: Thank you for your reminder. We have shared the data in figshare. The data from the public datasets are available in figshare. The data in this study can be found at DOI: 10.6084/m9.figshare.29299670. https://figshare.com/articles/online_resource/EOIdata_xlsx/29299670?file=55321631

(Page 14, Line 332-335)

3. Please amend your authorship list in your manuscript file to include author Lili Zhang,Yang Han, Yating Yang, Qian Sun, Zhuo Liu, Chenxi Wu, Shujuan Liang, Qianqian Jia.

4. Please amend your list of authors on the manuscript to ensure that each author is linked to an affiliation.

We note that you have included affiliation numbers 1,2,3 and 4 however only affiliations 1,2 and 3 have authors linked to them. Please amend affiliation 4 to link an author to it or remove if added in error.

Response: Thank you for your reminder. We are so sorry for our mistake. We have revised the manuscript, and uploaded the revised manuscript. ( Page 1, Line 3-8)

Attachment Submitted filename: Response to Reviewers.docx

# Peer review: Decision Letter 1

**DOI:** 10.1371/journal.pone.0355377.r003

**Authors:** Chong-Chi Chiu

7 Apr 2026

PONE-D-25-08783R1

Efficacy of ultrasound-guided external oblique intercostal plane block in laparoscopic common bile duct exploration: a randomized controlled study

PLOS One

Dear Dr.Jia,

Thank you for submitting your manuscript to PLOS ONE. After careful consideration, we feel that it has merit but does not fully meet PLOS ONE’s publication criteria as it currently stands. Therefore, we invite you to submit a revised version of the manuscript that addresses the points raised during the review process.

Please submit your revised manuscript by May 22 2026 11:59PM. If you will need more time than this to complete your revisions, please reply to this message or contact the journal office at plosone@plos.org. When you're ready to submit your revision, log on to https://www.editorialmanager.com/pone/ and select the 'Submissions Needing Revision' folder to locate your manuscript file.

Please include the following items when submitting your revised manuscript:

- A letter that responds to each point raised by the academic editor and reviewer(s). You should upload this letter as a separate file labeled 'Response to Reviewers'.
- A marked-up copy of your manuscript that highlights changes made to the original version. You should upload this as a separate file labeled 'Revised Manuscript with Track Changes'.
- An unmarked version of your revised paper without tracked changes. You should upload this as a separate file labeled 'Manuscript'.

If you would like to make changes to your financial disclosure, please include your updated statement in your cover letter. Guidelines for resubmitting your figure files are available below the reviewer comments at the end of this letter.

If applicable, we recommend that you deposit your laboratory protocols in protocols.io to enhance the reproducibility of your results. Protocols.io assigns your protocol its own identifier (DOI) so that it can be cited independently in the future. For instructions see: https://journals.plos.org/plosone/s/submission-guidelines#loc-laboratory-protocols. Additionally, PLOS ONE offers an option for publishing peer-reviewed Lab Protocol articles, which describe protocols hosted on protocols.io. Read more information on sharing protocols at https://plos.org/protocols?utm_medium=editorial-email&utm_source=authorletters&utm_campaign=protocols.

As the corresponding author, your ORCID iD is verified in the submission system and will appear in the published article. PLOS supports the use of ORCID, and we encourage all coauthors to register for an ORCID iD and use it as well. Please encourage your coauthors to verify their ORCID iD within the submission system before final acceptance, as unverified ORCID iDs will not appear in the published article. *Only*  the individual author can complete the verification step; PLOS staff *cannot*  verify ORCID iDs on behalf of authors.

We look forward to receiving your revised manuscript.

Kind regards,

Chong-Chi Chiu

Academic Editor

PLOS One

Journal Requirements:

If the reviewer comments include a recommendation to cite specific previously published works, please review and evaluate these publications to determine whether they are relevant and should be cited. There is no requirement to cite these works unless the editor has indicated otherwise.

Additional Editor Comments:

The reviewers have finished the review. Please revise your draft based on the suggestions and reply to the related questions.

Reviewers' comments:

Reviewer's Responses to Questions

**Comments to the Author**

1. If the authors have adequately addressed your comments raised in a previous round of review and you feel that this manuscript is now acceptable for publication, you may indicate that here to bypass the “Comments to the Author” section, enter your conflict of interest statement in the “Confidential to Editor” section, and submit your "Accept" recommendation.

Reviewer #1: (No Response)

Reviewer #2: All comments have been addressed

Reviewer #3: (No Response)

**********

2. Is the manuscript technically sound, and do the data support the conclusions?

The manuscript must describe a technically sound piece of scientific research with data that supports the conclusions. Experiments must have been conducted rigorously, with appropriate controls, replication, and sample sizes. The conclusions must be drawn appropriately based on the data presented.

Reviewer #1: Partly

Reviewer #2: Yes

Reviewer #3: Partly

**********

3. Has the statistical analysis been performed appropriately and rigorously?

Reviewer #1: Yes

Reviewer #2: Yes

Reviewer #3: Yes

**********

4. Have the authors made all data underlying the findings in their manuscript fully available?

The PLOS Data policy requires authors to make all data underlying the findings described in their manuscript fully available without restriction, with rare exception (please refer to the Data Availability Statement in the manuscript PDF file). The data should be provided as part of the manuscript or its supporting information, or deposited to a public repository. For example, in addition to summary statistics, the data points behind means, medians and variance measures should be available. If there are restrictions on publicly sharing data—e.g. participant privacy or use of data from a third party—those must be specified.

Reviewer #1: Yes

Reviewer #2: Yes

Reviewer #3: No

**********

5. Is the manuscript presented in an intelligible fashion and written in standard English?

PLOS ONE does not copyedit accepted manuscripts, so the language in submitted articles must be clear, correct, and unambiguous. Any typographical or grammatical errors should be corrected at revision, so please note any specific errors here.

Reviewer #1: Yes

Reviewer #2: Yes

Reviewer #3: Yes

**********

6. Review Comments to the Author

Please use the space provided to explain your answers to the questions above. You may also include additional comments for the author, including concerns about dual publication, research ethics, or publication ethics. (Please upload your review as an attachment if it exceeds 20,000 characters)

Reviewer #1: The analysis is much improved. The sample size issue was not addressed. It should at lease be presented as a convenience sample which cautions the interpretation of the p-values.

Reviewer #2: While this topic is potentially interesting, I have several concerns about the study design:

1. I do not think this clinical trial could be double-blind.

2. Please state how to randomly assign the patient.

3. Please add the inclusion and exclusion criteria in the method section, and please add more detail process about how to enroll study subjects

Reviewer #3: This manuscript reports a randomized controlled trial evaluating the efficacy of ultrasound-guided external oblique intercostal plane block (EOIPB) specifically in laparoscopic common bile duct exploration (LCBDE).

The reviewer has inquiries regarding the study design, and the selection of LCBDE as the procedure of choice for the trial:

1. The manuscript mentioned that a left-sided EOIPB was performed. What was the rationale for performing the block on the left side, when the target anatomy and incisions for LCBDE are typically on the right side?

2. LCBDE is often considered an adjunct to laparoscopic cholecystectomy, when indicated, and typically does not involved any additional incisions. Why was LCBDE selected for the study, and not the more prevalent/common cholecystectomy procedure. The Discussion section briefly mentions the locations of the incisions made for the procedure. Can the authors provide details on the size of the incisions, and whether fascial closure was performed at each of these incisions at the conclusion of the procedure, as the fascial closure can often be a significant contributor to postoperative pain.

3. Was the effects of the EOIPB limited to somatic pain, or were there effects on visceral pain as well?

4. The authors also mentioned that the effects of pneumoperitoneum can contribute to postoperative pain. What was the degree of pneumoperitoneum used for the LCBDE procedures, and what was the average operative time?

The reviewer also find that some essential information was missing from the manuscript, and further details on the execution of the trial is needed. Please adhere strictly to the CONSORT reporting guidelines for a complete list of the information needed:

1. How were participants identified for initial screening for in the trial? Additionally, more information is needed on how randomization was performed.

2. In this study, it is unclear how the patient can be blinded to the intervention, as the EOIPB is performed prior to anesthesia induction. Similarly, is the interventionalist performing the block separate from the anesthesiologist for the case, to ensure that the anesthesiologist is blinded to the assigned treatment? How did the anesthesiologist determine the dose of remifentanil to the administered intraoperatively, as this measure was an outcome reported for the study?

3. Postoperatively, was adjunctive pain control solely limited to the PCA? Or were there additional analgesics available as needed?

4. The EOIPB was performed in the operating room, prior to the induction of anesthesia. The Methods section also indicates that the block was tested 15 minutes later, again prior to anesthesia induction. Did this intervention lead to delays in the operating room? What was the mean duration from entry into the operating room to skin incision, and was it different between the intervention and control arms? Were there other post-procedural complications related to the EOIPB?

**********

7. PLOS authors have the option to publish the peer review history of their article (what does this mean?). If published, this will include your full peer review and any attached files.

If you choose “no”, your identity will remain anonymous but your review may still be made public.

**Do you want your identity to be public for this peer review?**  For information about this choice, including consent withdrawal, please see our Privacy Policy.

Reviewer #1: No

Reviewer #2: No

Reviewer #3: No

**********

[NOTE: If reviewer comments were submitted as an attachment file, they will be attached to this email and accessible via the submission site. Please log into your account, locate the manuscript record, and check for the action link "View Attachments". If this link does not appear, there are no attachment files.]

To ensure your figures meet our technical requirements, please review our figure guidelines: https://journals.plos.org/plosone/s/figures

You may also use PLOS’s free figure tool, NAAS, to help you prepare publication quality figures: https://journals.plos.org/plosone/s/figures#loc-tools-for-figure-preparation.

NAAS will assess whether your figures meet our technical requirements by comparing each figure against our figure specifications.

# Author comment: Author response to Decision Letter 2

**DOI:** 10.1371/journal.pone.0355377.r004

11 May 2026

Response to Reviewer 1

Reviewer #1: The analysis is much improved. The sample size issue was not addressed. It should at lease be presented as a convenience sample which cautions the interpretation of the p-values.

Response: We sincerely acknowledge this important comment. We have fully revised the Sample Size and Limitations sections.

We have supplemented the complete sample size calculation process based on an equivalence framework, including the predefined equivalence margins (Δ1 = 0.5 for VAS and Δ2 = 1 for PCA boluses), a two-sided significance level of α = 0.05, study power of 0.80, the standard deviation (SD) from pilot data, and an anticipated 10% dropout rate. This resulted in a target sample size of 60 patients (Page 7, Lines 193–199).

Although the sample size was calculated a priori, we acknowledge that the final sample.Remains relatively small and was derived from a single center, which limits generalizability. We have explicitly stated in the Limitations section that this study should be considered a convenience sample and that statistical significance and p-values should be interpreted with caution. The findings are hypothesis-generating and require validation in larger, multicenter trials (Page 16, Lines 395–399).

Response to Reviewer 2

Reviewer #2: While this topic is potentially interesting, I have several concerns about the study design:

1.I do not think this clinical trial could be double-blind.

Response: Thank you for this important reminder. We agree that full double-blinding is not feasible because EOIPB is an invasive regional block procedure; therefore, both patients and the anesthesiologist performing the block cannot be blinded to group allocation. We have clarified in the revised manuscript that this trial used assessor–statistician blinding:

Patients and the anesthesiologist performing EOIPB were aware of group assignment. Outcome assessors (VAS recording), anesthesiologists responsible for general anesthesia, and statisticians were fully blinded to group allocation throughout the study. This blinding approach is clearly described in the Methods section to avoid misinterpretation (Page 3, Lines 87–100).

2. Please state how to randomly assign the patient.

Response: Randomization was performed using a random number table prior to study initiation.

Eligible patients were randomized in a 1:1 ratio to the EOIPB group or the control group using sealed, sequentially numbered opaque envelopes. A research assistant (not involved in the study intervention, anesthesia management, or data collection) opened the envelopes and assigned patients to groups. The allocation was disclosed only to the physician responsible for performing EOIPB, while the anesthesiologists, statisticians, and observers remained blinded to group assignment. To ensure, allocation concealment, the observer remained outside the operating room during block performance and anesthesia induction. Full details have now been included in the Study Design section (Page 2, Lines 87–94).

3. Please add the inclusion and exclusion criteria in the method section, and please add more detail process about how to enroll study subjects.

Response: We have added the complete inclusion and exclusion criteria, as well as recruitment procedure, to the Study Design section:

Inclusion criteria were as follows: aged 18–65 years; American Society of Anesthesiologists (ASA) physical status I–II; diagnosis of choledocholithiasis and scheduled for elective laparoscopic common bile duct exploration (LCBDE); clear consciousness; ability to cooperate with postoperative pain scoring and follow-up; and provision of written informed consent. Exclusion criteria were as follows: history of liver disease, alcoholism, or drug abuse; mental illness; prior abdominal surgery or trauma; long-term opioid use; coagulation disorders; systemic or local infection at the injection site; conversion to open surgery during the procedure, or allergy to local anesthetics (Page 3, Lines 78–86).

Recruitment Procedure

Patients were screened preoperatively by a dedicated anesthesiologist. Those meeting the eligibility criteria were provided with a detailed explanation of the study purpose, procedures, and potential risks and benefits. After providing written informed consent, eligible patients were enrolled and randomized as described above. During the preoperative visit, baseline assessments and VAS/PCA training were conducted by the observer (Page 2, Lines 87–99).

Response to Reviewer 3

Reviewer #3: This manuscript reports a randomized controlled trial evaluating the efficacy of ultrasound-guided external oblique intercostal plane block (EOIPB) specifically in laparoscopic common bile duct exploration (LCBDE).

The reviewer has inquiries regarding the study design, and the selection of LCBDE as the procedure of choice for the trial:

1. The manuscript mentioned that a left-sided EOIPB was performed. What was the rationale for performing the block on the left side, when the target anatomy and incisions for LCBDE are typically on the right side?

Response: Thank you for your reminder. We sincerely apologize for this error. As shown in Figure 1, the procedure performed was a right-sided EOIPB. We have corrected this in the revised manuscript and uploaded the revised manuscript (Page 1, Line 22; Page 4, Line 117).

2.LCBDE is often considered an adjunct to laparoscopic cholecystectomy, when indicated, and typically does not involved any additional incisions. Why was LCBDE selected for the study, and not the more prevalent/common cholecystectomy procedure. The Discussion section briefly mentions the locations of the incisions made for the procedure. Can the authors provide details on the size of the incisions, and whether fascial closure was performed at each of these incisions at the conclusion of the procedure, as the fascial closure can often be a significant contributor to postoperative pain.

Response:

LCBDE was selected because it involves greater visceral traction, longer operative time, drainage tube insertion, and more intense postoperative pain (including somatic pain, visceral pain, and drainage tube-related irritation) compared with standard cholecystectomy. This model allows for better detection of analgesic effects of EOIPB.

The trocar sites and sizes were as follows: umbilicus (1.0–1.2 cm); subxiphoid region (0.5 cm); right subcostal region at the midclavicular line (1.5–2.0 cm); and right anterior axillary line (drain site, 0.5 cm).

Fascial closure was performed for all incisions at the end of surgery using standardized suturing techniques by surgeons from the same surgical team. These details have now been included to the Surgical Procedure section (Pages 6, Lines 156–171).

3.Was the effects of the EOIPB limited to somatic pain, or were there effects on visceral pain as well?

Response: Thank you for pointing this out. EOIPB mainly blocks somatic pain (skin, muscle, and parietal peritoneum) by interrupting this intercostal nerves and has no direct effect on visceral pain. However, it may indirectly improve overall pain perception through several mechanisms: reduced abdominal wall tenderness and guarding; attenuation of the stress response and secondary hyperalgesia; and decreased opioid consumption and related adverse effects. This distinction has been clarified in the Discussion (Page 15, Lines 371–373; Page 16, Lines 381–389).

4. The authors also mentioned that the effects of pneumoperitoneum can contribute to postoperative pain. What was the degree of pneumoperitoneum used for the LCBDE procedures, and what was the average operative time?

Response:

Intraoperative CO2 pneumoperitoneum was established at an insufflation rate of 2–4 L/min. Intra-abdominal pressure was maintained at 12–14 mmHg and dynamically adjusted by the anesthesiologist based on the patient's vital signs (blood pressure, heart rate, and airway pressure) and surgical requirements (Page 6, Lines 157–160).

The mean operative time was 82.40±17.93 min in the EOIPB group and 79.27±19.88 min in the control group (p>0.05) (Page 8-9, Table 1).

The reviewer also find that some essential information was missing from the manuscript, and further details on the execution of the trial is needed. Please adhere strictly to the CONSORT reporting guidelines for a complete list of the information needed:

1.How were participants identified for initial screening for in the trial? Additionally, more information is needed on how randomization was performed.

Response:

Initial screening: A dedicated anesthesiologist reviewed medical records, conducted a preoperative evaluation, assessed inclusion and exclusion criteria, and confirmed patient eligibility (Page 3, Lines 77–88).

Randomization: Randomization was performed using a random number table before study initiation. Eligible patients were randomized in a 1:1 to the EOIPB group or the control group using sealed, sequentially numbered opaque envelopes. A research assistant (not involved in the study intervention, anesthesia management, or data collection) opened the envelopes and assigned patients to groups. The allocation was disclosed only to the physician responsible for EOIPB, while the anesthesiologists, surgeons, and observers remained blinded to group assignment. To ensure allocation concealment, the observer remained outside the operating room during block performance and anesthesia induction. Full details have been included in the Study Design section (Page 3, Lines 87–92).

2.In this study, it is unclear how the patient can be blinded to the intervention, as the EOIPB is performed prior to anesthesia induction. Similarly, is the interventionalist performing the block separate from the anesthesiologist for the case, to ensure that the anesthesiologist is blinded to the assigned treatment? How did the anesthesiologist determine the dose of remifentanil to the administered intraoperatively, as this measure was an outcome reported for the study?

Response: Thank you for your reminder. We agree that full double-blinding is not feasible because EOIPB is an invasive regional block procedure; therefore, patients and the anesthesiologist performing the block cannot be blinded to group allocation. We have clearly stated in the revised manuscript that this trial employed assessor–statistician blinding:

Patients and the anesthesiologist performing EOIPB were aware of group assignment. Outcome assessors (VAS recording), anesthesiologists responsible for general anesthesia, and statisticians were fully blinded to group allocation throughout the study. This blinding design is clearly described in the Study Design section to avoid misinterpretation (Page 3-4, Lines 87–100).

Remifentanil was administered at a basal infusion rate of 0.1 ug/kg/min. The intraoperative infusion rate was adjusted based on heart rate (HR), mean arterial pressure (MAP), sweating, lacrimation, coughing, and body movement. If pain-related responses occurred, the infusion rate was increased by 0.05 ug/kg/min. If bradycardia or hypotension occurred, the infusion rate was decreased by 0.05 ug/kg/min. The bispectral index (BIS) was maintained between 40 and 60. These details are fully described in the Anesthesia management section (Page 5, Lines 137–141).

3. Postoperatively, was adjunctive pain control solely limited to the PCA? Or were there additional analgesics available as needed?

Response: Postoperative analgesia was primarily maintained with patient-controlled intravenous analgesia (PCIA). Patients with visual analog scale (VAS) scores ≥4 or those requesting additional analgesia received PCA as rescue analgesia. If analgesic efficacy was suboptimal, PCA administration could be repeated after 15 minutes following physician evaluation to ensure adequate pain control. No additional analgesics were administered within 24 h postoperatively. This has now been clarified in Section 2.4 (Postoperative Analgesia) (Page 5-6, Lines 146–151).

4. The EOIPB was performed in the operating room, prior to the induction of anesthesia. The Methods section also indicates that the block was tested 15 minutes later, again prior to anesthesia induction. Did this intervention lead to delays in the operating room? What was the mean duration from entry into the operating room to skin incision, and was it different between the intervention and control arms? Were there other post-procedural complications related to the EOIPB?

Response:

Thank you for pointing this out. We acknowledge that this aspect was not described in sufficient detail in the original manuscript. EOIPB and block assessment were performed in the pre-anesthesia room rather than in the operating room; therefore, no operating room delay occurred. To ensure adequate block onset, patients in the EOIPB group were transferred to the operating room 15 minutes earlier, as explained during the informed consent process. All patients were transferred from the pre-anesthesia room to the operating room (Page 4, Lines 101–106).

No EOIPB-related complications (bleeding, infection, hematoma, pneumothorax, or local anesthetic toxicity) were observed in any patient. These data have been added to the Results section (Page 14, Lines 322–324).

Attachment Submitted filename: Response to Reviewers.doc

# Peer review: Decision Letter 2

**DOI:** 10.1371/journal.pone.0355377.r005

**Authors:** Chong-Chi Chiu

10 Jun 2026

PONE-D-25-08783R2

Efficacy of ultrasound-guided external oblique intercostal plane block in laparoscopic common bile duct exploration: a randomized controlled study

PLOS One

Dear Dr. Jia,

Thank you for submitting your manuscript to PLOS ONE. After careful consideration, we feel that it has merit but does not fully meet PLOS ONE’s publication criteria as it currently stands. Therefore, we invite you to submit a revised version of the manuscript that addresses the points raised during the review process.

Please submit your revised manuscript by Jul 25 2026 11:59PM. If you will need more time than this to complete your revisions, please reply to this message or contact the journal office at plosone@plos.org. When you're ready to submit your revision, log on to https://www.editorialmanager.com/pone/ and select the 'Submissions Needing Revision' folder to locate your manuscript file.

Please include the following items when submitting your revised manuscript:

- A letter that responds to each point raised by the academic editor and reviewer(s). You should upload this letter as a separate file labeled 'Response to Reviewers'.
- A marked-up copy of your manuscript that highlights changes made to the original version. You should upload this as a separate file labeled 'Revised Manuscript with Track Changes'.
- An unmarked version of your revised paper without tracked changes. You should upload this as a separate file labeled 'Manuscript'.

If you would like to make changes to your financial disclosure, please include your updated statement in your cover letter. Guidelines for resubmitting your figure files are available below the reviewer comments at the end of this letter.

If applicable, we recommend that you deposit your laboratory protocols in protocols.io to enhance the reproducibility of your results. Protocols.io assigns your protocol its own identifier (DOI) so that it can be cited independently in the future. For instructions see: https://journals.plos.org/plosone/s/submission-guidelines#loc-laboratory-protocols. Additionally, PLOS ONE offers an option for publishing peer-reviewed Lab Protocol articles, which describe protocols hosted on protocols.io. Read more information on sharing protocols at https://plos.org/protocols?utm_medium=editorial-email&utm_source=authorletters&utm_campaign=protocols.

As the corresponding author, your ORCID iD is verified in the submission system and will appear in the published article. PLOS supports the use of ORCID, and we encourage all coauthors to register for an ORCID iD and use it as well. Please encourage your coauthors to verify their ORCID iD within the submission system before final acceptance, as unverified ORCID iDs will not appear in the published article. *Only*  the individual author can complete the verification step; PLOS staff *cannot*  verify ORCID iDs on behalf of authors.

We look forward to receiving your revised manuscript.

Kind regards,

Chong-Chi Chiu

Academic Editor

PLOS One

Journal Requirements:

1. If the reviewer comments include a recommendation to cite specific previously published works, please review and evaluate these publications to determine whether they are relevant and should be cited. There is no requirement to cite these works unless the editor has indicated otherwise.

2. Please review your reference list to ensure that it is complete and correct. If you have cited papers that have been retracted, please include the rationale for doing so in the manuscript text, or remove these references and replace them with relevant current references. Any changes to the reference list should be mentioned in the rebuttal letter that accompanies your revised manuscript. If you need to cite a retracted article, indicate the article’s retracted status in the References list and also include a citation and full reference for the retraction notice.

Additional Editor Comments:

The reviewers have completed their review of your manuscript. Please revise the article according to their comments and suggestions.

Reviewers' comments:

Reviewer's Responses to Questions

**Comments to the Author**

1. If the authors have adequately addressed your comments raised in a previous round of review and you feel that this manuscript is now acceptable for publication, you may indicate that here to bypass the “Comments to the Author” section, enter your conflict of interest statement in the “Confidential to Editor” section, and submit your "Accept" recommendation.

Reviewer #1: All comments have been addressed

Reviewer #2: All comments have been addressed

Reviewer #3: All comments have been addressed

**********

2. Is the manuscript technically sound, and do the data support the conclusions?

The manuscript must describe a technically sound piece of scientific research with data that supports the conclusions. Experiments must have been conducted rigorously, with appropriate controls, replication, and sample sizes. The conclusions must be drawn appropriately based on the data presented.

Reviewer #1: (No Response)

Reviewer #2: Yes

Reviewer #3: Yes

**********

3. Has the statistical analysis been performed appropriately and rigorously?

Reviewer #1: (No Response)

Reviewer #2: Yes

Reviewer #3: Yes

**********

4. Have the authors made all data underlying the findings in their manuscript fully available?

The PLOS Data policy requires authors to make all data underlying the findings described in their manuscript fully available without restriction, with rare exception (please refer to the Data Availability Statement in the manuscript PDF file). The data should be provided as part of the manuscript or its supporting information, or deposited to a public repository. For example, in addition to summary statistics, the data points behind means, medians and variance measures should be available. If there are restrictions on publicly sharing data—e.g. participant privacy or use of data from a third party—those must be specified.

Reviewer #1: (No Response)

Reviewer #2: Yes

Reviewer #3: Yes

**********

5. Is the manuscript presented in an intelligible fashion and written in standard English?

PLOS ONE does not copyedit accepted manuscripts, so the language in submitted articles must be clear, correct, and unambiguous. Any typographical or grammatical errors should be corrected at revision, so please note any specific errors here.

Reviewer #1: (No Response)

Reviewer #2: Yes

Reviewer #3: Yes

**********

6. Review Comments to the Author

Please use the space provided to explain your answers to the questions above. You may also include additional comments for the author, including concerns about dual publication, research ethics, or publication ethics. (Please upload your review as an attachment if it exceeds 20,000 characters)

Reviewer #1: (No Response)

Reviewer #2: The authors have responded appropriately to the reviewers’ comments, and the manuscript has been revised accordingly. The quality and clarity of the manuscript have improved substantially after revision. Overall, the authors have adequately addressed the major concerns raised during the review process, and I have no further comments or suggestions.

Reviewer #3: The manuscript has improved substantially following revision, and the authors have adequately addressed the major concerns raised by the reviewers.

However, several areas would benefit from additional clarification to further strengthen the manuscript. More detailed demographic and clinical information regarding the patient populations in both study groups would be valuable. Specifically, the authors should report the underlying pathology necessitating laparoscopic common bile duct exploration (LCBDE), including the presence of acute cholecystitis, biliary pancreatitis, cholangitis, or other relevant conditions. Additional information regarding the etiology of common bile duct obstruction (e.g., choledocholithiasis, benign stricture, malignancy, or other causes) would also improve the characterization of the study cohort. Furthermore, reporting preoperative pain severity or symptom burden would help establish baseline comparability between groups.

The manuscript would also benefit from greater detail regarding the operative techniques employed during LCBDE. Specifically, the authors should clarify whether stone extraction was performed using basket retrieval, balloon catheter extraction, choledochoscopy-assisted techniques, sphincterotomy, or choledochotomy with direct stone removal. Information regarding bile duct closure methods, if applicable, would also be helpful. Finally, operative duration should be reported and compared between groups, as it represents an important procedural outcome and may influence perioperative results.

**********

7. PLOS authors have the option to publish the peer review history of their article (what does this mean?). If published, this will include your full peer review and any attached files.

If you choose “no”, your identity will remain anonymous but your review may still be made public.

**Do you want your identity to be public for this peer review?**  For information about this choice, including consent withdrawal, please see our Privacy Policy.

Reviewer #1: No

Reviewer #2: No

Reviewer #3: No

**********

[NOTE: If reviewer comments were submitted as an attachment file, they will be attached to this email and accessible via the submission site. Please log into your account, locate the manuscript record, and check for the action link "View Attachments". If this link does not appear, there are no attachment files.]

To ensure your figures meet our technical requirements, please review our figure guidelines: https://journals.plos.org/plosone/s/figures

You may also use PLOS’s free figure tool, NAAS, to help you prepare publication quality figures: https://journals.plos.org/plosone/s/figures#loc-tools-for-figure-preparation.

NAAS will assess whether your figures meet our technical requirements by comparing each figure against our figure specifications.

# Author comment: Author response to Decision Letter 3

**DOI:** 10.1371/journal.pone.0355377.r006

24 Jun 2026

Response to Reviewer 3

Comment 1: More detailed demographic and clinical information regarding the patient populations in both study groups would be valuable. Specifically, the authors should report the underlying pathology necessitating laparoscopic common bile duct exploration (LCBDE), including the presence of acute cholecystitis, biliary pancreatitis, cholangitis, or other relevant conditions. Additional information regarding the etiology of common bile duct obstruction (e.g., choledocholithiasis, benign stricture, malignancy, or other causes) would also improve the characterization of the study cohort. Furthermore, reporting preoperative pain severity or symptom burden would help establish baseline comparability between groups.

Response:

Thank you for this important suggestion. We agree that detailed clinical characteristics can better characterize the study population and help verify baseline comparability between groups.

In response to this comment, we have supplemented the relevant information in the Study Design and Results sections of the revised manuscript:

We added a detailed classification of the primary underlying diseases for all enrolled patients, including acute cholecystitis, acute cholangitis, biliary pancreatitis and simple cholelithiasis with choledocholithiasis , and compared their distribution between the two groups (Page 8-9, Table1).

We clarified the etiology of common bile duct obstruction and specified that all included patients were diagnosed with choledocholithiasis (Page 3, Line 79); no patients with benign biliary stricture or malignant biliary obstruction were enrolled in this trial , and this information has now been explicitly stated in the manuscript (Page 3, Lines 82-84).

Due to an oversight in the study design, preoperative pain scores and symptom burden were not recorded, as the potential impact of these variables on the baseline comparability between groups was not considered.We acknowledge that this may limit the ability to establish baseline comparability between groups. This limitation has been added to the Limitations section of the revised manuscript (Page 16, Lines 402-404).

Comment 2: The manuscript would also benefit from greater detail regarding the operative techniques employed during LCBDE. Specifically, the authors should clarify whether stone extraction was performed using basket retrieval, balloon catheter extraction, choledochoscopy-assisted techniques, sphincterotomy, or choledochotomy with direct stone removal. Information regarding bile duct closure methods, if applicable, would also be helpful. Finally, operative duration should be reported and compared between groups, as it represents an important procedural outcome and may influence perioperative results.

Response:

Thank you for this valuable suggestion regarding the reporting of surgical technical details. In response, we have extended the Surgical Procedures section to provide a more detailed and standardized description of the laproscopic common bile duct exploration (LCBDE) procedure.

1)Surgical techniques: We added a detailed description of the LCBDE procedure in the Surgical Procedures section. All patients underwent laparoscopic choledochotomy combined with choledochoscopy-assisted stone extraction. For most calculi, choledochoscope-guided basket retrieval was the preferred method of stone extraction. For small, sandy, or residual calculi, balloon catheter extraction was used as an adjunctive technique to facilitate biliary tract clearance. No endoscopic sphincterotomy was performed in any of the enrolled patients (Page 5-6, Lines 148-158).

2)Bile duct closure: After complete stone clearance, primary closure of the common bile duct was performed in all patients without T-tube drainage. Details regarding the suture material and suturing technique have also been added to the manuscript (Page 5-6, Lines 158-162).

3)Operative duration: We recorded and analyzed the total operative time both groups in the Results section. No significant difference in operative duration was observed between the two groups (P>0.05), suggesting that operative time was unlikely to have confounded perioperative pain outcomes or other study endpoints(Page 9, Table 1).

Attachment Submitted filename: Response_to_Reviewers_auresp_3.docx

# Peer review: Decision Letter 3

**DOI:** 10.1371/journal.pone.0355377.r007

**Authors:** Chong-Chi Chiu

21 Jul 2026

Efficacy of ultrasound-guided external oblique intercostal plane block in laparoscopic common bile duct exploration: a randomized controlled study

PONE-D-25-08783R3

Dear Dr. Jia,

We’re pleased to inform you that your manuscript has been judged scientifically suitable for publication and will be formally accepted for publication once it meets all outstanding technical requirements.

Within one week, you’ll receive an e-mail detailing the required amendments. When these have been addressed, you’ll receive a formal acceptance letter and your manuscript will be scheduled for publication.

An invoice will be generated when your article is formally accepted. Please note, if your institution has a publishing partnership with PLOS and your article meets the relevant criteria, all or part of your publication costs will be covered. Please make sure your user information is up-to-date by logging into Editorial Manager at Editorial Manager® and clicking the ‘Update My Information' link at the top of the page. For questions related to billing, please contact billing support.

If your institution or institutions have a press office, please notify them about your upcoming paper to help maximize its impact. If they’ll be preparing press materials, please inform our press team as soon as possible -- no later than 48 hours after receiving the formal acceptance. Your manuscript will remain under strict press embargo until 2 pm Eastern Time on the date of publication. For more information, please contact onepress@plos.org.

Kind regards,

Chong-Chi Chiu

Academic Editor

PLOS One

Reviewers' comments:

Reviewer's Responses to Questions

**Comments to the Author**

1. If the authors have adequately addressed your comments raised in a previous round of review and you feel that this manuscript is now acceptable for publication, you may indicate that here to bypass the “Comments to the Author” section, enter your conflict of interest statement in the “Confidential to Editor” section, and submit your "Accept" recommendation.

Reviewer #1: All comments have been addressed

Reviewer #2: All comments have been addressed

Reviewer #3: All comments have been addressed

**********

2. Is the manuscript technically sound, and do the data support the conclusions?

The manuscript must describe a technically sound piece of scientific research with data that supports the conclusions. Experiments must have been conducted rigorously, with appropriate controls, replication, and sample sizes. The conclusions must be drawn appropriately based on the data presented.

Reviewer #1: (No Response)

Reviewer #2: Yes

Reviewer #3: Yes

**********

3. Has the statistical analysis been performed appropriately and rigorously?

Reviewer #1: (No Response)

Reviewer #2: Yes

Reviewer #3: Yes

**********

4. Have the authors made all data underlying the findings in their manuscript fully available?

The PLOS Data policy requires authors to make all data underlying the findings described in their manuscript fully available without restriction, with rare exception (please refer to the Data Availability Statement in the manuscript PDF file). The data should be provided as part of the manuscript or its supporting information, or deposited to a public repository. For example, in addition to summary statistics, the data points behind means, medians and variance measures should be available. If there are restrictions on publicly sharing data—e.g. participant privacy or use of data from a third party—those must be specified.

Reviewer #1: (No Response)

Reviewer #2: Yes

Reviewer #3: Yes

**********

5. Is the manuscript presented in an intelligible fashion and written in standard English?

PLOS ONE does not copyedit accepted manuscripts, so the language in submitted articles must be clear, correct, and unambiguous. Any typographical or grammatical errors should be corrected at revision, so please note any specific errors here.

Reviewer #1: (No Response)

Reviewer #2: Yes

Reviewer #3: Yes

**********

6. Review Comments to the Author

Please use the space provided to explain your answers to the questions above. You may also include additional comments for the author, including concerns about dual publication, research ethics, or publication ethics. (Please upload your review as an attachment if it exceeds 20,000 characters)

Reviewer #1: (No Response)

Reviewer #2: The manuscript is acceptable in its current form. The authors have adequately addressed the reviewers’ comments and have revised the manuscript accordingly.

Reviewer #3: (No Response)

**********

7. PLOS authors have the option to publish the peer review history of their article (what does this mean?). If published, this will include your full peer review and any attached files.

If you choose “no”, your identity will remain anonymous but your review may still be made public.

**Do you want your identity to be public for this peer review?**  For information about this choice, including consent withdrawal, please see our Privacy Policy.

Reviewer #1: No

Reviewer #2: No

Reviewer #3: No

**********

# Editor report: Acceptance letter

**DOI:** 10.1371/journal.pone.0355377.r008

**Authors:** Chong-Chi Chiu

PONE-D-25-08783R3

PLOS One

Dear Dr. Jia,

I'm pleased to inform you that your manuscript has been deemed suitable for publication in PLOS One. Congratulations! Your manuscript is now being handed over to our production team.

At this stage, our production department will prepare your paper for publication. This includes ensuring the following:

* All references, tables, and figures are properly cited

* All relevant supporting information is included in the manuscript submission,

* There are no issues that prevent the paper from being properly typeset

You will receive further instructions from the production team, including instructions on how to review your proof when it is ready. Please keep in mind that we are working through a large volume of accepted articles, so please give us a few days to review your paper and let you know the next and final steps.

Lastly, if your institution or institutions have a press office, please let them know about your upcoming paper now to help maximize its impact. If they'll be preparing press materials, please inform our press team within the next 48 hours. Your manuscript will remain under strict press embargo until 2 pm Eastern Time on the date of publication. For more information, please contact onepress@plos.org.

You will receive an invoice from PLOS for your publication fee after your manuscript has reached the completed accept phase. If you receive an email requesting payment before acceptance or for any other service, this may be a phishing scheme. Learn how to identify phishing emails and protect your accounts at https://explore.plos.org/phishing.

If we can help with anything else, please email us at customercare@plos.org.

Thank you for submitting your work to PLOS ONE and supporting open access.

Kind regards,

PLOS ONE Editorial Office Staff

on behalf of

Professor Chong-Chi Chiu

Academic Editor

PLOS One
